# A Self‐immolative Molecular Beacon for Amplified Nucleic Acid Detection**

**DOI:** 10.1002/chem.202102600

**Published:** 2021-09-13

**Authors:** Magdalena Roth, Oliver Seitz

**Affiliations:** ^1^ Institute of Chemistry Humboldt-Universität zu Berlin Brook-Taylor-Str. 2 12489 Berlin Germany

**Keywords:** fluorescent probes, photo chemistry, oligonucleotides, RNA recognition, templated chemistry

## Abstract

Fluorogenic hybridization probes allow the detection of RNA and DNA sequences in homogeneous solution. Typically, one target molecule activates the fluorescence of a single probe molecule. This limits the sensitivity of nucleic acid detection. Herein, we report a self‐immolative molecular beacon (iMB) that escapes the one‐target/one‐probe paradigm. The iMB probe includes a photoreductively cleavable *N*‐alkyl‐picolinium (NAP) linkage within the loop region. A fluorophore at the 5’‐end serves, on the one hand, as a reporter group and, on the other hand, as a photosensitizer of a NAP‐linker cleavage reaction. In the absence of target, the iMB adopts a hairpin shape. Quencher groups prevent photo‐induced cleavage. The iMB opens upon hybridization with a target, and both fluorescent emission as well as photo‐reductive cleavage of the NAP linker can occur. In contrast to previous chemical amplification reactions, iMBs are unimolecular probes that undergo cleavage leading to products that have lower target affinity than the probes before reaction. Aided by catalysis, the method allowed the detection of 5 pm RNA target within 100 min.

## Graphical abstract

**The target as catalyst**: A new category of nucleic acid‐templated reaction is introduced. Binding of a self‐immolative molecular beacon (iMB) to a DNA/RNA target can trigger a photocatalyzed cleavage reaction. The products have lower target affinity than the iMB before the reaction. This enables the target to act as a catalyst providing chemical amplification of signals. The unimolecular iMB probe allowed detection of 5 pm RNA within 100 min.

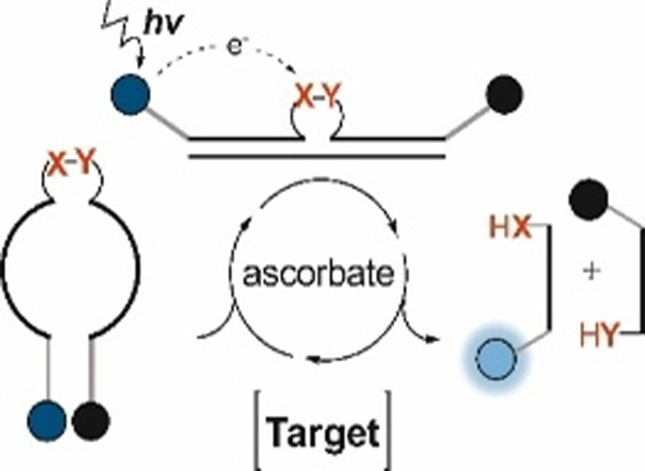

Probe molecules that fluoresce upon recognition of specific nucleic acid targets are invaluable tools for applications in molecular diagnostics.^[1]^ However, without added enzymes, the probe molecules remain bound to the target and, as a result, the fluorescence enhancement is low when probes are present in large excess of the target; for example, when the concentration of the nucleic acid target in a biological sample is low.

Chemical amplification methods provide a solution to the one‐target‐activates‐one‐probe issue.^[2]^ Initial work in the field was focused on template‐controlled ligation reactions (Figure 1A);^[3]^ however, ligation is plagued by product inhibition. The introduction of destabilizing linkers^[4]^ or unpaired nucleotides^[5]^ helped reduce the stability of the formed probe‐template duplex, but the ligation products still had higher template affinity than the probes before ligation. To enable turnover of template, reactions must be performed at high reactant access in the early phase of the ligation when probes before reaction can compete for template binding. Because ligation yields remain low, a relatively small number of product molecules must be detected against the background of a high excess of reactant molecules. It must be taken into account that the excess cannot be increased arbitrarily, otherwise template‐independent reactions will occur at high concentrations. A significant improvement was achieved with templated functional group interconversions (Figure 1B), in which the number of nucleotides in the reactant and product molecules remains unchanged. Noteworthy examples include reaction systems that induced transfer or cleavage of fluorophores,^[6]^ or removal of fluorescence quencher groups (such as azide, tetrazine or vinyl ether groups) from fluorophores.^[7]^ To promote turnover, such reactions are performed under conditions of dynamic strand exchange. At low template amounts, inevitably, only a small fraction of the reactant molecules binds, reactions become slow and long reaction times (>2 h) will be needed to accumulate signal. A third category of nucleic acid templated reactions involves the adjacent hybridization of oligonucleotide‐ligand conjugates to stabilize metal complexes that catalyze the conversion of small molecule substrates without product inhibition (Figure 1C).[^7d^, ^8^] Though high sensitivities in the femtomolar range have been reported for example for DNA‐templated Pd catalysis,^[8a]^ the tolerance to oxygen and typical catalyst poisons such as thiols has not been studied.

**Figure 1:**
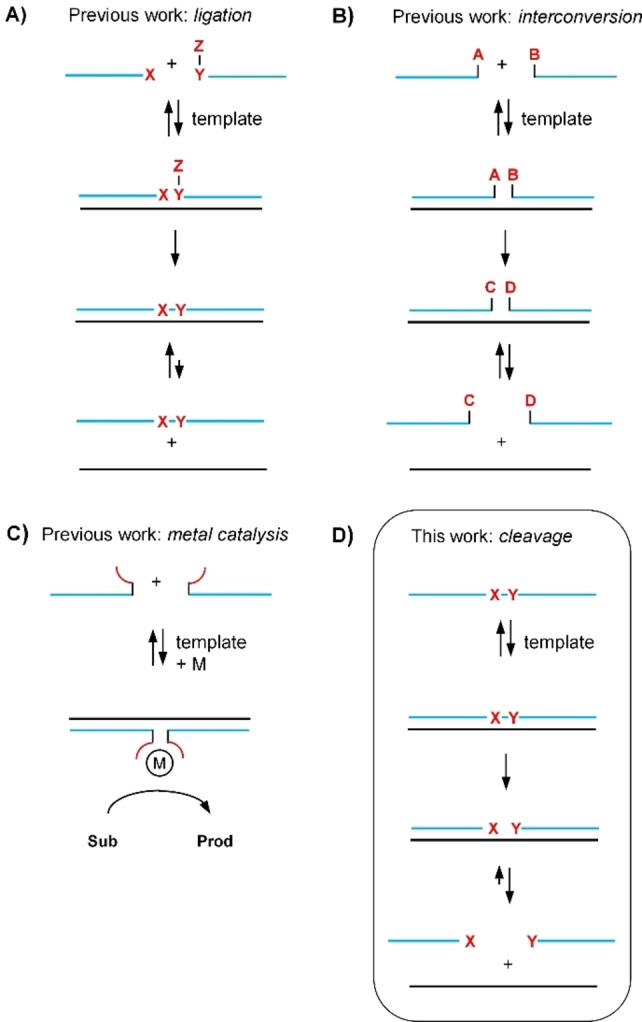
Categories of nucleic‐acid‐templated chemical reactions. A) ligation reactions; B) functional group interconversions; C) metal‐catalyzed reactions and D) cleavage reactions. A, B, C, D, X, Y, Z=functional groups; M=metal; Sub=substrate; Prod=product.

We envisioned a new category of nucleic acid‐templated reactions; systems that induce cleavage within the main chain of an oligonucleotide probe (Figure 1D). We assumed that cleavage should lead to product fragments that have a lower affinity for the target than the probe before reaction. Under these conditions, multiple reactions can proceed on a single target, hence enabling a catalytic chemical signal amplification. In contrast to previously reported reaction systems, which involve the interplay and optimization of two or even more functionalized oligonucleotides, a single probe should be sufficient. Ideally, target engagement would trigger the self‐destruction of the unimolecular probe. Interestingly, oligonucleotide cleavage by protein or nucleic acid enzymes is the basis of an increasing number of assays aiming for isothermal signal amplification.^[9]^ Such systems depend on correct folding of the enzymatically active units and are, therefore, affected by solvents, detergents and variations of ionic strength. Chemical methods tolerate a wider range of conditions. However, while chemical alternatives for ligase enzymes do exist,^[3]^ a detection method providing a chemical alternative to cleavage reactions based on DNAzymes and nicking enzymes is lacking.

Herein, we introduce self‐immolative molecular beacon (iMB) probes, which include a photocleavable linker unit within the loop region (X−Y in Figure 2). The cleavage reaction is catalyzed by a fluorophore such as the coumarin **1** which serves on the one hand as a reporter group and on the other hand as a photosensitizer of the cleavage reaction. Analogously to conventional molecular beacons,[^1c^, ^1d^, ^1e^, ^1f^] the iMB probe is envisioned to adopt a hairpin shape in absence of target. This positions a quencher in vicinity of the fluorophore. Accordingly, fluorescence and also photosensitizing activity remain low. Target binding separates the fluorophore from the quencher (→**2**). This restores fluorescence and activates the fluorophore‘s ability to photosensitize cleavage. The ternary complex formed upon cleavage has lower target affinity than the iMB probe prior cleavage. The product complex **3** can dissociate and the target will be able to act as a catalyst with little interference from product inhibition commonly observed in known templated reactions.

**Figure 2:**
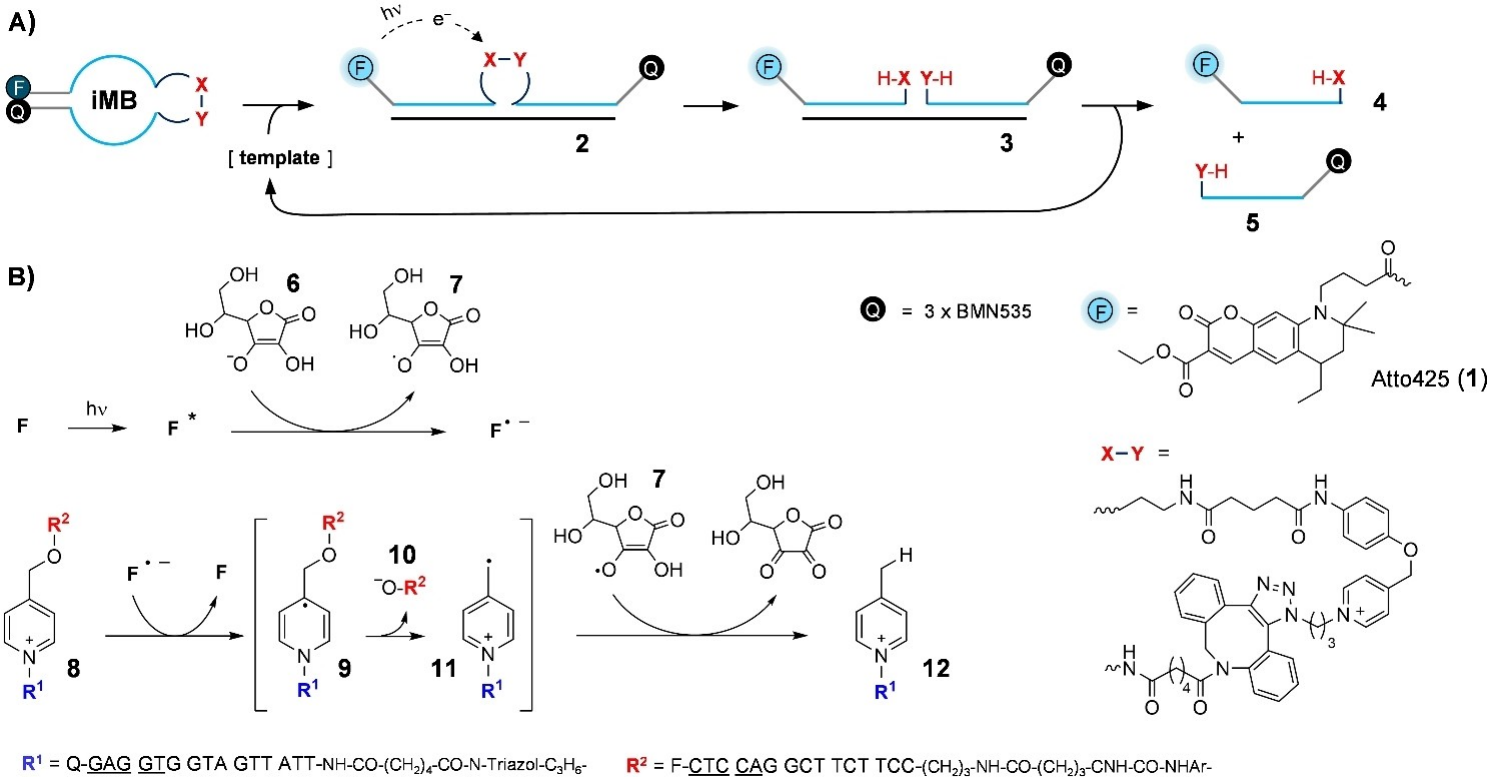
A) Concept of fluorescence signaling by an iMB. A scissile unit X−Y (e. g., the depicted *N*‐alkylpicolinium (NAP)‐phenol ether) enables dye (F)‐sensitized iMB cleavage under the control of the nucleic acid target. The products **4** and **5** dissociate from the template (RNA target) and a new iMB can bind. B) Proposed mechanism of photoreductive NAP‐phenol ether cleavage.^[10]^ Underlined nucleotides indicate the MB stem unit.

The photocleavable linker is the key unit of the iMB design. In the pursuit of a suitable linker, we were fascinated by a report from Winssinger,^[10]^ who described exceptionally fast photocleavage reactions of the *N*‐alkyl‐picolinium (NAP) protecting group.^[11]^ First reported by Falvey,^[12]^ [Ru(bpy)_3_]^2+^ complexes were used as efficient photocatalysts of the DNA template‐controlled cleavage of NAP‐caged fluorophores. Falvey also reported liberation of amino acids and phosphates from NAP esters using high wavelength laser dyes acting as photosensitizers.^[13]^ The metal‐free photorelease reactions were performed in organic solvents. Intrigued by the prospect of combining fluorescence read‐out and photocatalysis within a single dye we investigated whether metal‐free photocleavage reactions also proceed in aqueous buffer solutions. In test reactions, we dissolved a NAP‐protected phenol in an aqueous buffer containing 10 mm ascorbate as reductant and the ATTO425 (**1**) dye as photosensitizer (Figure S11 in the Supporting Information). Gratifyingly, irradiation with a 455 nm blue LED induced efficient cleavage, also in the aqueous environment. As previously suggested, a likely cleavage mechanism involves a single electron transfer step from ascorbate **6** to the dye's excited state and from there to the electrophilic NAP group **8** (Figure 2B).^[14]^ The resulting pyridine radical **9** then expels the phenolate leaving group **10**. The reduction is complete when the *N*‐alkylpicolinium radical **11** formed upon cleavage abstracts hydrogen from ascorbate or the ascorbate radical.

To introduce the NAP linker in self‐immolative molecular beacons (iMB, Figure 1B), we used a postsynthetic route to avoid exposure of the base labile NAP‐phenyl ether to conditions of ammonia cleavage. The bifunctional handle **13** (Figure 3) was employed in coupling reactions with two commercially available oligonucleotides (Scheme S2). In this proof‐of‐concept study, the iMB was designed for recognition of a 20‐mer RNA segment transcribed from the b2a2 BCR‐ABL fusion gene. A pentamer stem (60 % GC content) was used to bring the ATTO425 dye into the vicinity of the quencher unit, which was comprised of three BMN535 dyes to allow efficient quenching of fluorescent emission. Fluorescence spectra of the iMB before and after hybridization with an RNA target confirmed that the iMB behaved like a “traditional” MB probe (Figures 4A and S12). Despite the disruption of contiguous base pairing, introduced by the NAP linker, the hybridization with one equivalent RNA target induced an opening of the hairpin, as inferred from the eightfold increase of fluorescence at 485 nm.

**Figure 3:**
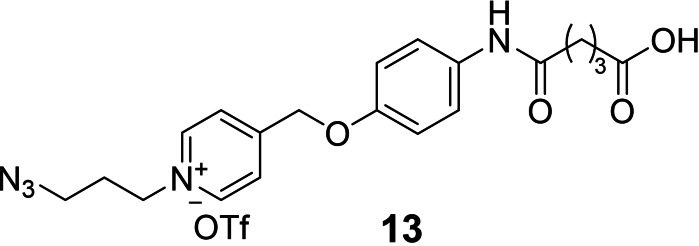
Chemical structure of N‐alkylpicolinium‐phenylether conjugate used for synthesis of iMB probes.

**Figure 4:**
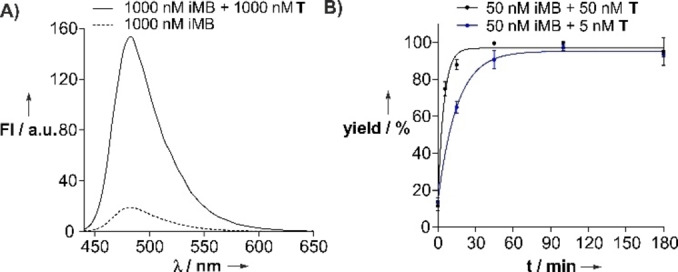
A) Fluorescence spectra of iMB in absence and presence of target (5’‐CCAUCAAUAAGGAAGAAGCC‐3’). B) Time course of the templated photocleavage of iMB. Conditions: 10 mM NaH_2_PO_4_, 100 mM NaCl, 2.5 mM MgCl2, 5 mM ascorbate, pH 7.4, *T*=37 °C; for B): irradiation at *λ*=455 nm, yield determined by UPLC‐FL (Figure S14A).

Next, we examined the photo‐induced self‐immolation of the iMB by means of fluorescence detected UPLC analysis. In absence of target, no new product appeared after three hours irradiation time (Figure S14B). By contrast, the addition of one equivalent target to 50 nm iMB triggered a rapid photo cleavage (Figures 4B and S14 A). Assuming pseudo‐first‐order kinetics, we determined a reaction half‐time *t*_1/2_ of 3.3 min (Figure 4B). Importantly, a surprisingly rapid cleavage was also obtained when the reaction was performed in presence of 0.1 equivalents template. After 45 min, cleavage had occurred in 90 % yield. This suggests that the products of the photo‐induced cleavage reaction are readily displaced from the template by excess iMB probe.

To probe the target affinity of the iMB photo cleavage products we performed UV melting experiments (Table 1, Figures S17 and S18) with fragments **4 a** and **5 a** (Figure 5), which contain the characteristic linker features (e. g., DBCO unit, alkyl spacers). The melting temperatures *T*_M_ provided by the fragments, alone or in combination, were 10–20 °C lower than the *T*_M_=56 °C of the intact iMB‐target duplex. We infer: self‐immolation of the iMB does indeed lead to a loss of target affinity. Interestingly, a comparison of the *T*_M_ values of the iMB in absence and presence of target suggested a surprisingly high stability of the iMB hairpin structure (*T*_M_ (iMB)=57 °C vs. *T*_M_ (iMB ⋅ T)=56 °C). However, compared to the iMB ⋅ T system the iMB melting curve (Figure S17) shows a rather shallow sigmoidal transition indicative of low cooperativity. This might facilitate hairpin opening.

**Table 1:** Melting temperatures of complexes formed upon hybridization of target T with the iMB and its fragments.^[a]^

Complex	iMB	iMB ⋅ T	**4 a** ⋅ **5 a** ⋅ T	**4 a** ⋅ T	**5 a** ⋅ T	T
T_M_/°C	57 °C	56 °C	43 °C	48 °C	38 °C	39 °C

[a] Conditions: 500 nm oligonucleotide, PBS (10 mm NaH_2_PO_4_, 100 mm NaCl, 2.5 mm MgCl_2_, 5 mm ascorbate, pH 7.4, F: ATTO425; Q: 3xBMN535. Melting curves see Figures S15–S18.

**Figure 5:**
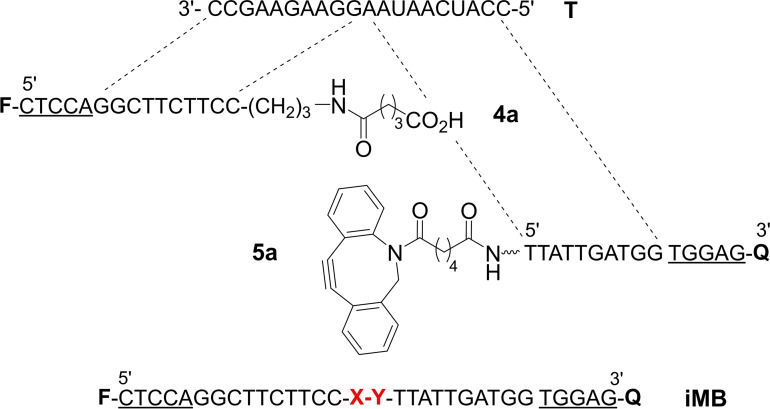
Structure of nucleic acid components used for measurements of melting temparatures.

Encouraged by the loss of target affinity upon photo‐self‐immolation, we analyzed fluorescence signaling after irradiation of the iMB in presence of substoichiometric target amounts. With 50 nm iMB, the presence of 5 nm (0.1 equiv) target was signalled by an 150 % increase of fluorescence at 485 nm (Figure 6). Signaling was fast. A 100 % signal increase was obtained after 15 min. Without photoirradiation the uncleaved iMB afforded a 52 % signal increase, which corresponds to the signal expected for “traditional” uncleavable MB probe with similar fluorescence turn‐on characteristics (Table 2). This signal increase afforded by the uncleaved probe falls to 11 or 4 % with 0.02 or 0.005 equiv. target, respectively. With 125 and 64 % change of signal, much higher signaling was observed after 220 min irradiation. Of note, marked 30 % signal increases were obtained already after 45 min or less (Figure 6). These experiments point to the advantage of self‐immolating MB probes: a single target molecule can activate the fluorescence of many probe molecules.

**Figure 6:**
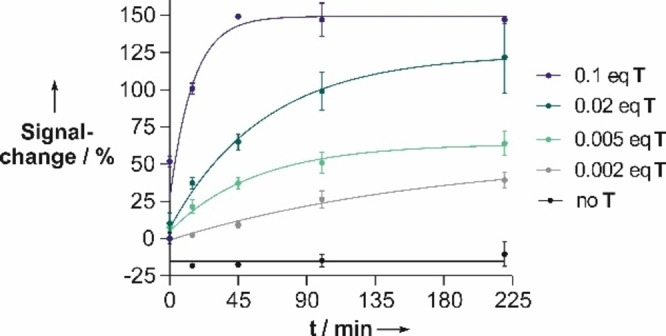
Time course of fluorescence signaling upon iMB photocleavage of substoichiometric target T. Conditions: 50 nM iMB, *c*(T) as indicated in buffer (10 mm NaH_2_PO_4_, 100 mm NaCl, 2.5 mm MgCl_2_, 5 mm ascorbate, pH 7.4), *T*=37 °C, irradiation at 455 nm. Signal change=(*F*/*F*_0_−1)×100 %, *F*_0_, *F* are the fluorescence intensity at 485 nm (*λ*_ex_=430 nm) before or after addition of target.

**Table 2:** Signal enhancement prior to and after photoirradiation of iMB.

Equivalents	Signal change^[a]^ [%]	
of T	without cleavage	after 220 min photo‐irradiation
0.1	52±3.5	147±2
0.02	11±3.1	124±44
0.01	7±1.4	100±40
0.005	4±2.3	64±8
0.002	not detectable	39±5

[a] Signal change [%]=(*F*/*F*_0_−1)×100 %. Conditions: see Figure 3.

The analysis of template‐induced fluorescence signaling upon photo‐cleavage shown in Figure 6 revealed that the maximum signal enhancement (Figures 4A and S12) was not reached. Control experiments showed that the ATTO425 coumarin dye suffers from photo bleaching (Figure S13). Though bleaching reduces the achievable signal enhancement, it helps to reduce the background signal in absence of template.

Next, we investigated the limit of detection (LOD). After 100 min photoirradiation in PBS buffer, 100 pm target provided a 27 % signal enhancement (Figure 7A). Tween20 was added to the buffer. This detergent presumably not only hinders adsorption or unwanted aggregation but potentially serves also as an anti‐fade agent. With this adjustment, 5 pm RNA target afforded a 52±8 % signal increase after 100 min (compared to 23±4 % without target; Tables S2 and S3).

**Figure 7:**
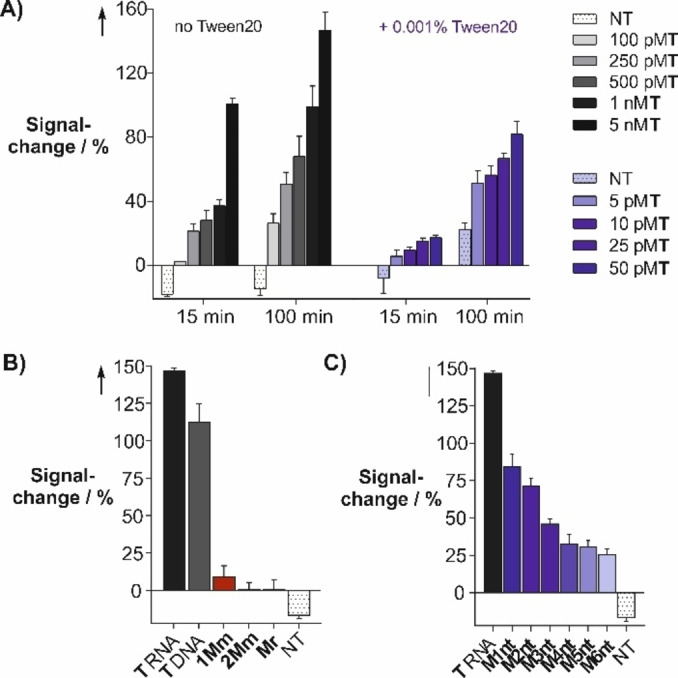
Fluorescence signal change from iMB after A) 15 min/100 min photoirradiation in presence of 0.1–0.0001 equiv. target T. Dependence of signaling from iMB on B) the target sequence and C) the number of gap nucleotides opposite the NAP linkage after 220 min. Conditions: 50 nm iMB, 5 nM (or lower where indicated) target, 10 mm NaH_2_PO_4_, 100 mm NaCl, 2.5 mm MgCl_2_, 5 mm ascorbate, 0.001 % Tween20 (if added), pH 7.4, 37 °C, irradiation at 455 nm. Signal change=(*F*/*F*_0_−1)×100 %.

In subsequent experiments, we evaluated the sequence specificity of the iMB probe (Figure 7B). For this purpose, the iMB probe was incubated with a single mismatched RNA target (**1Mm**), a target with two mismatched nucleotides (**2Mm**) and a random target (**Mr**). In addition, a perfectly complementary DNA target (**M_DNA_**) was added. After 100 min photoirradiation, the signal remained virtually unchanged when the iMB was incubated with the random sequence control or the target containing two mismatched nucleotides. Incubation with the single mismatched target resulted in a negligible signal change. As expected, the complementary DNA target promoted photo cleavage.

In further control experiments, the targeted segments were separated by an increasing number of gap nucleotides (Figure 7C). Targets including one or two nucleotide insertions still provided for marked signal enhancements. Signaling gradually decreased as the distance between targeted segments was increased. Apparently, the cleavable linker within the iMB loop portion induces a certain degree of tolerance to nucleotide insertions between the targeted segments. We attribute this behavior to a bivalency effect. The spacer‐separated oligonucleotide segments within the iMB loop resemble a spacer‐linked bivalent receptor, which recognizes a bivalent ligand (i. e., the target). As previously reported, bivalent interactions loose strength as the distance between the receptor‐ligand pairs increases.^[15]^ The highest affinity should be provided by a target that enables coaxial stacking of the iMB loop segments and indeed, the highest fluorescent signal was obtained with a “gapless” target. Despite the tolerance for nucleotide insertions, we wish to note that a target that involves two 10 nt segments separated by fewer than six nucleotides should still be unique.

In conclusion, we have developed a photochemical method for signal‐amplified nucleic acid detection. In contrast to previously reported chemical methods, the self‐immolative molecular beacon (iMB) approach is based on template‐controlled cleavage leading to products that have lower template affinity than the probe before reaction. The iMB probe studied provided a 5 pm limit for the detection of an RNA target in buffer. First experiments in cell lysate and RNA extract (Figures S20 and S21) indicate that photo‐induced self‐immolation increased the sensitivity of the MB probe also in complex matrices.

It is instructive to compare the results of our study with previous template‐controlled photochemical reactions. Winssinger developed a reaction system involving four probes; two Ru^2+^‐containing oligonucleotide strands and two PNA strands. Triggered by only 8 pm template, a combination of hybridization chain reactions and [Ru]^2+^‐mediated photocleavage of NAP‐quenched PNA‐fluorophore conjugates provided a ≈10 % signal change after 3 h of reaction.^[16]^ The iMB probe required only 100 min to signal 5 pm of target by means of 30 % fluorescence increase relative to the no‐template reaction. Mokhir used DNA‐photosensitizer conjugates to form singlet oxygen to induce the cleavage of 9‐alkoxyanthracen‐linked fluorophore‐DNA conjugates. A 10 pm detection limit was reported for a 30‐min reaction involving the concerted action of four dye‐labeled oligonucleotide conjugates.^[17]^ In contrast to this set‐up and all previously reported templated chemistries, our method uses a single probe. Unimolecular hybridization probes such as molecular beacons have been widely applied.

With the current iMB system, fluorescence‐based nucleic acid detection is limited by a relatively modest fluorescence enhancement (eightfold) upon iMB opening, and photobleaching. Alternative photosensitizer schemes might allow for improvements. Based on data from Falvey et al., an exergonic photo‐reductive cleavage of NAP linkers succeeded with coumarin and BODIPY dyes having oxidation potentials of photosensitizers *E*_ox_=1.01–1.22 V and energies of the sensitizer singlet state *E*_0.0_=2.32–2.65 eV.^[13]^ Other dyes such as carbopyronines might be applicable.^[18]^ In analogy to work published from Falvey and Winssinger, iMB probes might also involve a quenched [Ru(bpy)_3_]^2+^ dye for photo‐catalyzing the cleavage of NAP‐caged coumarin linkers.[^10a^, ^14^, ^18d^]

The data obtained in this study prove the feasibility of nucleic acid cleavage as a new category of DNA/RNA‐templated reaction. We have shown that a self‐immolating option improves the sensitivity of DNA molecular beacons. The concept is probably not restricted to the photo‐reductive cleavage of NAP‐type linkers. Other photo‐triggered cleavage reactions are known. With this and the alternative labeling schemes discussed above, we consider the iMB principle as a new reaction paradigm for DNA/RNA detection chemistries. It seems likely that further improvements allowing fluorescence detection of sub‐picomolar targets are within reach.

## Experimental Section

Experimental details can be found in the Supporting Information.

## Conflict of interest

The authors declare no conflict of interest.

## Supporting information

As a service to our authors and readers, this journal provides supporting information supplied by the authors. Such materials are peer reviewed and may be re‐organized for online delivery, but are not copy‐edited or typeset. Technical support issues arising from supporting information (other than missing files) should be addressed to the authors.

Supporting Information
