# Supplementary material for: A Self‐immolative Molecular Beacon for Amplified Nucleic Acid Detection
Source: Chemistry. 2021 Sep 13;27(57):14189–94. doi: 10.1002/chem.202102600 (PMC8597011; doi:10.1002/chem.202102600)
Supplement: Supplementary file 1 — Supporting Information [file CHEM-27-14189-s001.pdf]

Chemistry–A European Journal

Supporting Information

A Self-immolative Molecular Beacon for Amplified Nucleic Acid Detection**

Magdalena Roth and Oliver Seitz*

1 Materials and Methods

1.1 Materials

Modified oligonucleotides (**S9** and **S11**) were purchased from Biomers (Ulm, Germany). Chemicals for the purification of DNA such as acetonitrile (Water content < 30 ppm), isopropanol and ethanol were purchased from Roth (Karlsruhe, Germany). All other chemicals were purchased from Acros, Sigma Aldrich, Fluka, TCI or abcr and were used without further purification. Water was purified with an Astacus Milli-Q Ultra Pure Water Purification System from membraPure GmbH (Bodenheim, Germany).

1.2 Instruments and Methods

Semipreparative HPLC of oligonucleotides was performed on a 1105 HPLC System from Gilson equipped with an UV/Vis-detector (260 nm detection wavelength) and a Waters X-Bridge BEH 130 C18 (10 x 150 mm), 5 µm column at 55 °C. The flow rate was set to 7 mL/min. A binary mixture of A (0.1 M triethylammoniumacetate at pH = 7.3) and B (acetonitrile) was used as mobile phase.

Analytical HPLC was performed on a 1105 HPLC System from Gilson equipped with an UV/Vis-detector (260 nm detection wavelength) and a Waters X-Bridge BEH 130 C18 (4.6 x 250 mm), 5 µm column at 55 °C. The flow rate was set to 1.5 mL/min. A binary mixture of A (0.1 M triethylammoniumacetate at pH = 7.3) and B (acetonitrile) was used as mobile phase.

MALDI-TOF mass spectrometry was performed on a Shimadzu Axima Confidence (positive linear mode). As an excitation source, a nitrogen Laser was used at λ = 337 nm. HPA (0.15 M 2,3,4-trihydroxyacetophenone in MeCN/Water (1:1 [v/v] with 0.5 M diammonium-citrate) was used as a Matrix.

Fluorescence measurements were performed on a Cary Varian Eclipse (Agilent technologies) spectrometer equipped with a peltier block by using either suprasil ultra micro quartz cuvettes (100 µL, d = 1 cm) or suprasil quartz cuvettes (1 mL, d = 1 cm) at either 25 °C or 37 °C. All measurements were performed in degassed phosphate buffer (10 mM NaH_2PO_4, 100 mM NaCl, pH = 7.4) containing $MgCl_2$, sodium ascorbate and Tween20 as indicated (ex: 430 nm, slit: 5 nm, em: 485 nm, slit: 5 nm). All measurements were done in triplicate.

For UPLC analysis a Waters ACQUITY was equipped with an ACQUITY UPLC TUV detector as well as a fluorescence detector (ex: 430 nm, em: 490 nm). An AQCUITY UPLC BEH oligonucleotide C18 (130 A, 1.7 µm, 2.1 · 50 mm) column was used at a flow rate of 0.5 mL/min at 50 °C. As a mobile phase, a binary mixture of A (0.1 M triethylammoniumacetate at pH = 7.3) and B (acetonitrile) was used.

Photo-induced cleavage was performed in F-96 well plates from PerkinElmer sealed with Greiner EASYseal clear A5596 – 100EA (Sigma-Aldrich) seal-film. The well plate was irradiated with a collimated LED-light placed approx. 15 cm above the plate (455 nm, 1 W: Thorlabs, part. Number M455L2-C2 – www.thorlabs.com) using 75%-85% brightness for set times. The well-plate was cooled/heated with the aid of a custom-made-aluminum-block equipped with a Julabo F250 temperature controller (JULABO GmbH).

UV melting curves were measured at 260 nm using a JASCO spectrometer equipped with a peltier block in UV quartz cuvettes (d = 1 cm), V = 1mL, c = 500 nM. The oligonucleotides were mixed in a 1:1 ratio. The melting curves were recorded at a rate of 0.5 °C/min (20 °C – 90 °C). This was repeated 4x per melting curve. The absorption was normalized and the melting temperature was calculated from the first derivative.

Cell lysate: To release the adherent HEK293WT cells from the surface of the culture dish, the medium was removed and washed once with PBS. Subsequently 0.25% trypsin EDTA (ThermoFischer) was added and incubated for 3-5 min at 37 °C. The cells were detached from the culture dish and the trypsin reaction was stopped by adding full medium (DMEM, 10% FBS (serum), 1x streptomycin/penicillin). The suspension was centrifuged (200 x g, 5 min). The supernatant was discarded. Next, the cells were resuspended in medium and counted (ThermoFischer Countess). The desired number of cells were centrifuged and washed with PBS buffer. The cells were then redissolved in TRIS buffer and proteinase K (3-4 h, 50 °C, 250 µg/mL final conc.) was added. After centrifugation ((200 x g, 5 min, RT), the desired lysate was filtrated before use.

RNA-extract: a cell pellet from 437 mio. HEK293WT cells was dissolved in 15 mL trizol (cooled). Cells were subsequently lysed by pipetting up and down until completely dissolved. Next, 3 mL chloroform was added and the mixture was agitated for 15 sec. The mix was incubated for 3 min at room temperature followed by centrifugation for 15 min at 13.000 rpm (4 °C). The upper layer containing the extracted RNA was carefully pipetted into a sterile falcon tube. One volume-part (pipetted volume of extracted RNA) of cold iPrOH was added along with 1/10-Volume parts of 3 M Na-Acetate. After precipitation overnight, the falcon was centrifuged at 4 °C for 15 min at 4000 rpm. The iPrOH was decanted and the precipitate was washed 10 x with cooled 70% EtOH-solution. The pellet was dried over argon and then resuspended in DEPC-Water. The concentration as well as the purity were determined via Nanodrop: Determined ratios (absorbance maxima):260/280: 1.98; 260/230: 2.3.

NMR spectra were recorded on a BRUKER Avance II (400 MHz) using $CDCl_3$, CD_3OD or DMSO-d6 as solvent, and were referenced with respective residual ^{1}H-solvent peaks of $CHCl_3$ (δ = 7.26 ppm), CHD_2OD (δ = 3.31 ppm), and DMSO (δ = 2.50 ppm) and ^{13}C-solvent peaks of $CDCl_3$ (δ = 77.0 ppm), CD_3OD (δ = 49.05 ppm), and DMSO-d6 (δ = 39.43 ppm).

LC-MS spectra were recorded using a Waters ACQUITY UPLC coupled with a QDa Mass Spectrometer System (electrospray ionization (ESI)) operating in positive mode on a Waters Acquity UPLC BEH C18 1-7 µm column.

2 Synthesis and Characterization

2.1 Synthesis of the *N*-Alkyl-picolinium cleavage linker (NAP-CL)

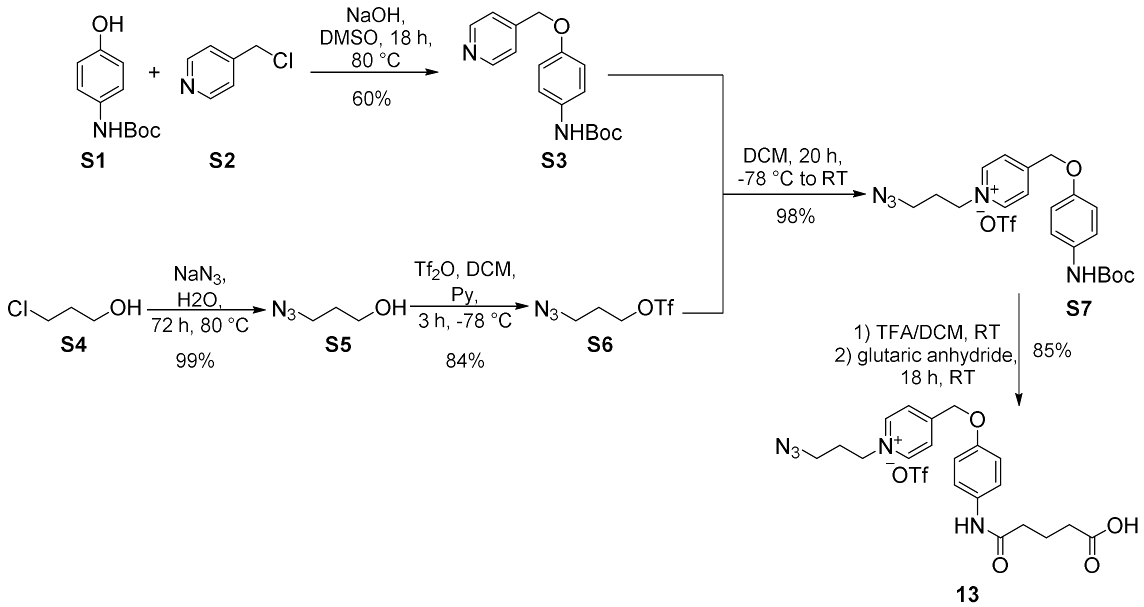

Scheme S1. Synthesis of the NAP linker **13**

2.1.1 4-*tert.*-Butyloxycarbonylamidophenyl-picolyl ether (**S3**)

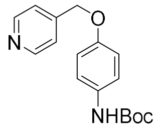

In a two-neck flask under Ar, 4-*N*-Boc-aminophenol (**S1**) (5.97 g, 28.0 mmol, 1.0 eq) was dissolved in 60 mL dry DMSO. After heating to 50 °C, NaOH (2.80 g, 70 mmol, 2.5 eq) was added. The suspension was stirred at this temperature for 1 h. Next, 3-chloro-methylpyridine (**S2**) (4.64 g, 28.0 mmol, 1.0 eq) was added within 2 h. The reaction mixture was heated to 80 °C and stirred for 18 h. After cooling to room temperature, the mixture was poured onto 500 mL of ice and extracted from EtOAc (10 x 300 mL). The combined organic layers were washed with water (10 x 300 mL) and dried over $MgSO_4$. The solvent was removed in vacuo and the crude product was purified via flash-chromatography (DCM/EtOAc 4:1) to yield **S3** (4.93 g, 60%) as a white solid. **^{1}H-NMR** (500 MHz, $CDCl_3$): δ[ppm] = 8.60 (dd, *J* = 4.5, 1.5 Hz, 2H), 7.35 (d, *J* = 6.1 Hz, 2H), 7.28 (d, *J* = 8.3 Hz, 2H), 6.89 – 6.87 (m, 2H), 6.51 (s, 1H), 5.05 (s, 2H), 1.50 (s, 9H). **^{13}C-NMR** (126 MHz, $CDCl_3$): δ[ppm] = 154.2, 153.2, 149.9, 146.6, 132.4, 121.1, 120.6, 115.3, 68.6, 28.5. LC-MS (ESI) RT = 2.4 min (3-80% B in 4 min). m/z calcd for $C_{17}H_{20}N_2O_3$ +H^+: 301.15 $[M+H]^+$, found: 301.28 $[M+H]^+$.

3-Azido-1-propanol (**S5**)[1]

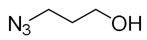

In a 100 mL flask, 3-Chloro-1-propanol (**S4**) (1.89 g, 20.0 mmol, 1.0 eq) was dissolved in 40 mL ddH_2O. Sodium azide (2.60 g, 40.0 mmol, 2.0 eq) was added successively. The reaction mixture was heated to 80 °C and stirred for 72 h. After cooling to RT, the crude mixture was extracted with DCM (4 x 70 mL). The combined organic layers were dried over $MgSO_4$. The solvent was

removed in vacuo (35 °C, max. 150 mbar) to yield the product (**S5**, 2.02 g, 99%) without further purification as a clear colorless oil. The NMR-data is in agreement with the given literature-data[1]. **^{1}H-NMR** (500 MHz, $CDCl_3$): δ[ppm] = 3.73 (t, *J* = 6.0 Hz, 2H), 3.44 (t, *J* = 6.6 Hz, 2H), 1.98 (s, 1H), 1.91 – 1.72 (m, 2H). **^{13}C-NMR** (126 MHz, $CDCl_3$): δ[ppm] = 60.0, 48.5, 31.2.

2.1.2 3-Azido-1-triflyl-propanol (**S6**)[2]

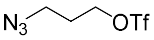

S6

In a 100 mL flask under Ar, 3-Azido-1-propanol (**S5**) (2.00 g, 20.0 mmol, 1.0 eq) was dissolved in 45 mL DCM and cooled to 0 °C. At this temperature, pyridine (2.62 g, 24.0 mmol, 1.2 eq) was added and subsequently cooled to –78 °C. Next, Triflic-anhydride (6.23 g, 22.0 mmol, 1.1 eq) was added within 20 min. The reaction mixture was stirred for another 3 h at –78 °C and then warmed to RT. 30 mL sat. NH_4Cl was added to quench the reaction followed by an extraction with EtOAc (3 x 70 mL). The combined organic layers were dried over $MgSO_4$. The solvent was removed in vacuo and the crude was purified with flash-chromatography (Hexane/EtOAc 10:1) to yield the product (**S6,** 3.91 g, 84%) as a clear colorless oil. The NMR-Data is in agreement with the given literature-data[2]. **^{1}H-NMR** (500 MHz, $CDCl_3$): δ[ppm] = 4.63 (t, *J* = 6.0 Hz, 2H), 3.52 (t, *J* = 6.4 Hz, 2H), 2.08 – 2.06 (m, 2H). **^{13}C-NMR** (126 MHz, $CDCl_3$): δ[ppm] = 73.9, 46.9, 28.9.

2.1.3 4-*tert*.-Butyloxycarbonylamidophenyl *N*-azidopropylpicolinium ether (**S7**)

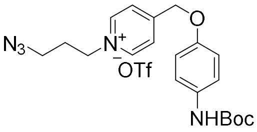

S7

In a 250 mL two-neck flask under Ar, **S3** (600 mg, 2.0 mmol, 1.0 eq) was dissolved in 170 mL dry DCM. The solution was cooled to –78 °C and 3-azido-1-triflyl-propanol (**S6**) (653 mg, 2.80 mmol, 1.4 eq) was added successively. The reaction mixture was warmed to RT and stirred for another 20 h. The solvent was removed in vacuo and the crude product was purified via flash-chromatography (DCM/MeOH 10:1) to yield **S7** (1.05 g, 99%) as a white solid. **^{1}H-NMR** (500 MHz, CD_3OD): δ[ppm] = 8.97 (d, *J* = 6.8 Hz, 2H), 8.19 (d, *J* = 6.7 Hz, 2H), 7.37 (d, *J* = 8.9 Hz, 2H), 7.11 – 6.76 (m, 2H), 5.45 (s, 2H), 4.73 (t, *J* = 7.2 Hz, 2H), 3.55 (s, 2H), 3.37 (s, 2H), 2.43 – 2.14 (m, 2H), 1.53 (s, 9H). **^{13}C-NMR** (126 MHz, CD_3OD): δ[ppm] = 160.4, 155.6, 154.5, 145.9, 134.9, 126.5, 123.1, 121.8, 120.5, 116.2, 80.8, 68.6, 60.11, 31.2, 28.7. LC-MS (ESI) RT = 2.7 min (3-80% B in 4 min). m/z calcd for $C_{21}F_3H_{26}N_5O_6S$: 533.15, found: 384.24 $[M\text{-}OTf]^+$.

2.1.4 NAP-CL (**13**)

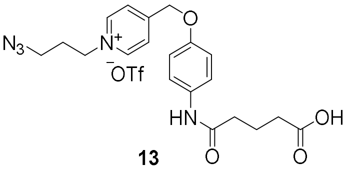

13

To a 50 mL flask under Ar, **S7** (1.05 g, 1.98 mmol, 1.0 eq) was added and dissolved in DCM/TFA (1:1, [v/v] 16 mL) and stirred for 1 h at RT. The deprotection was followed via LC-MS. After quantitative deprotection, the solvent mixture was evaporated by means of an argon stream, washed 5 x with DCM and dried under reduced pressure for 30 min. The residue was dissolved in water (10 mL) and sat. $NaHCO_3$ was added until the solution reached pH = 8.0 and a white solid precipitated. The precipitate was dissolved in EtOAc followed by an extraction with EtOAc (5 x 60 mL). The combined organic layers were dried over $MgSO_4$ and the solvent was reduced in vacuo. The aniline derivative was used without further purification.

To a 250 mL flask under Ar, aniline intermediate (745 mg, 1.72 mmol, 1.0 eq) was added and dissolved in dry EtOAc (90 mL). Next, glutaric anhydride (255 mg, 2.24 mmol, 1.3 eq) was added. The reaction mixture was heated to 50 °C until the suspension was completely dissolved. The solution was then stirred at RT for 18 h. The formed precipitate was filtrated, washed with cold EtOAc and subsequently dried at reduced pressure. The product (**13**, 800 mg, 85%) was obtained as a pale-yellow solid and used without further purification. **^{1}H-NMR** (500 MHz, CD_3OD): δ[ppm] = 8.96 (d, *J* = 6.8 Hz, 2H), 8.17 (d, *J* = 6.6 Hz, 2H), 7.59 – 7.39 (m, 2H), 7.09 – 6.92 (m, 2H), 5.45 (s, 2H), 4.71 (t, *J* = 7.2 Hz, 2H), 3.51 (t, *J* = 6.3 Hz, 2H), 2.40 (dt, *J* = 12.7, 7.3 Hz, 4H), 2.33 – 2.17 (s, 2H), 1.98 (dd, *J* = 16.0, 8.6 Hz, 3H), 1.24 (t, *J* = 7.1 Hz, 1H). **^{13}C-NMR** (126 MHz, CD_3OD): δ[ppm] = 179.9, 173.6, 160.2, 155.5, 145.9, 134.3, 126.5, 123.2, 116.1, 68.6, 60.1, 36.8, 34.2, 31.2, 22.2, 20.9. LC-MS (ESI) RT = 1.8 min (3-80% B in 4 min). m/z calcd for $C_{21}F_3H_{24}N_5O_7S$: 547.13 g/mol, found: 398.38 $[M\text{-}OTf]^+$.

2.2 NMR-Spectra

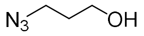

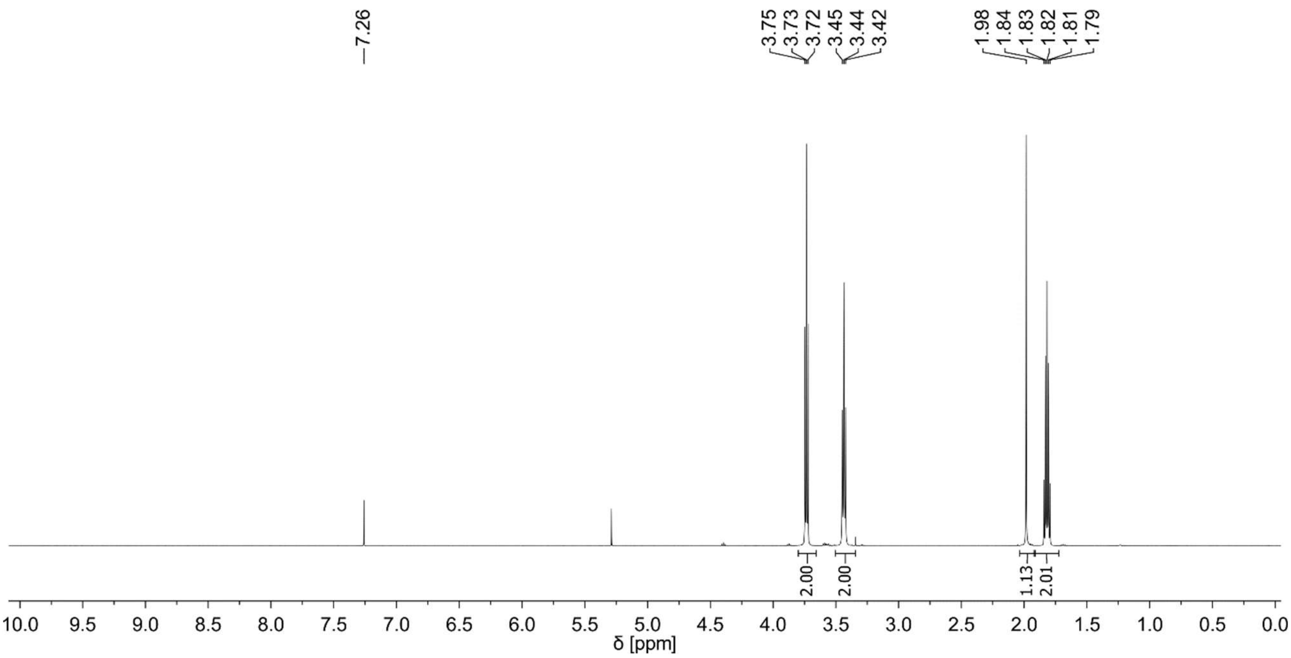

Figure S1. **^{1}H-NMR** (500 MHz, $CDCl_3$) of **S5.**

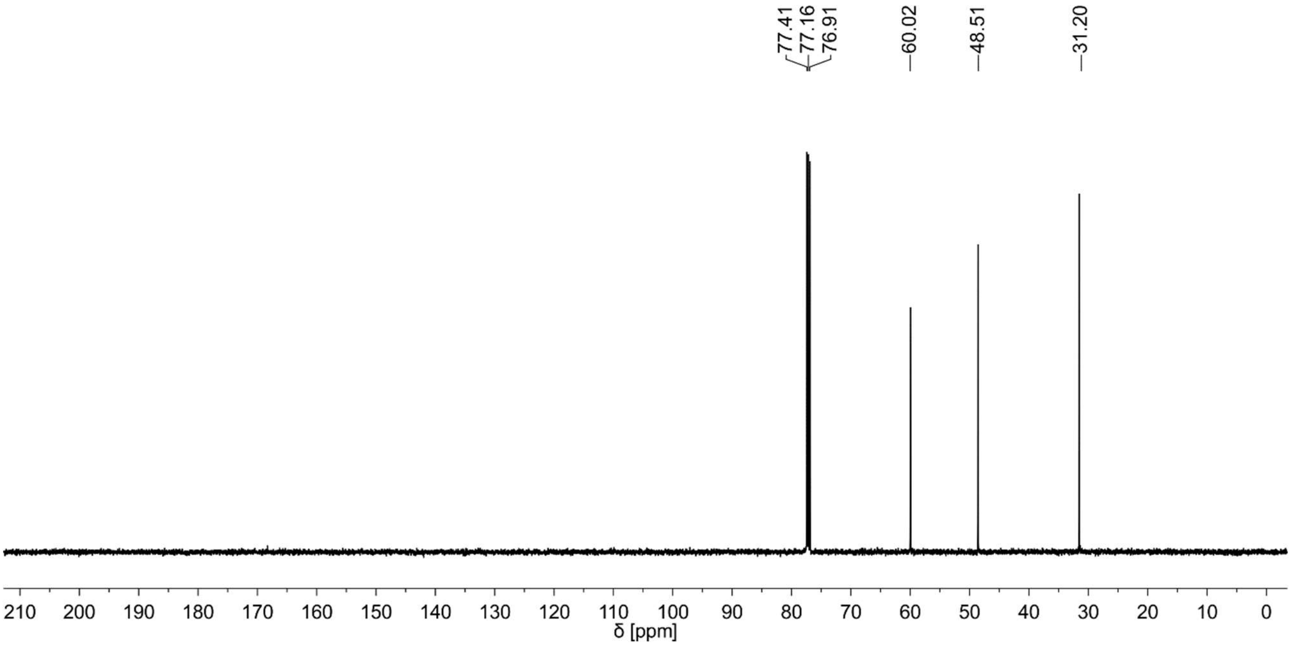

Figure S2. **^{13}C-NMR** (126 MHz, $CDCl_3$) of **S5.**

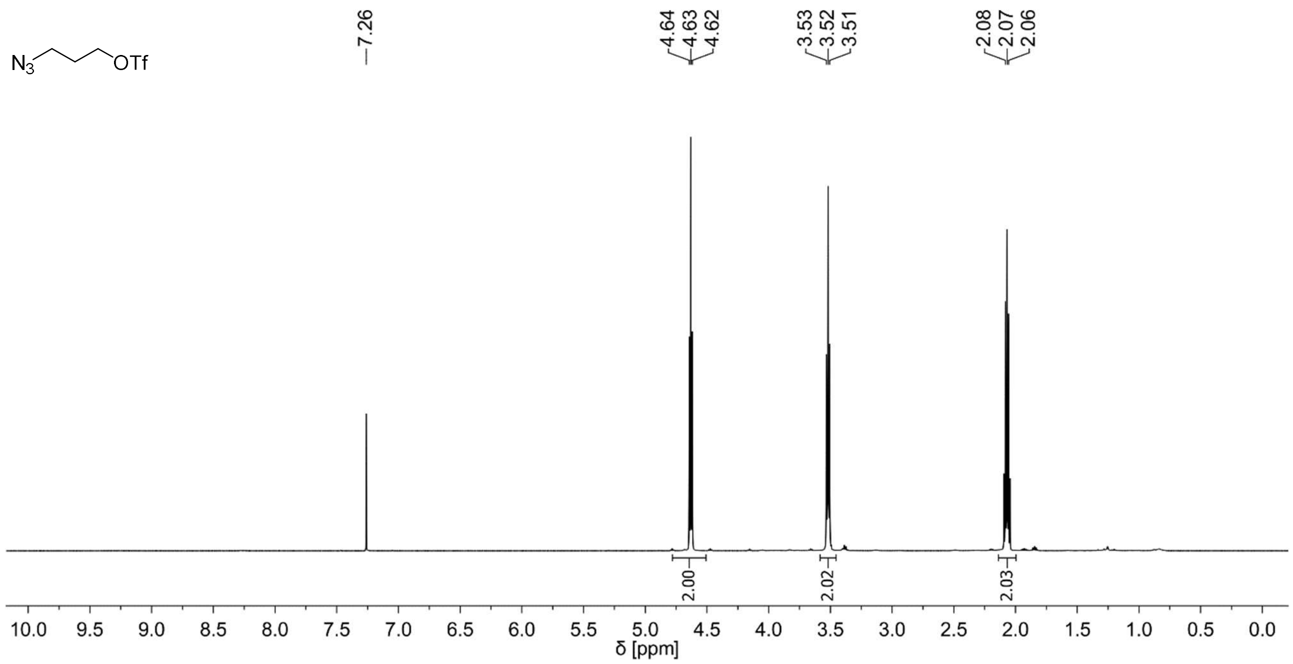

Figure S3. [1]**H-NMR** (500 MHz, $CDCl_3$) of **S6.**

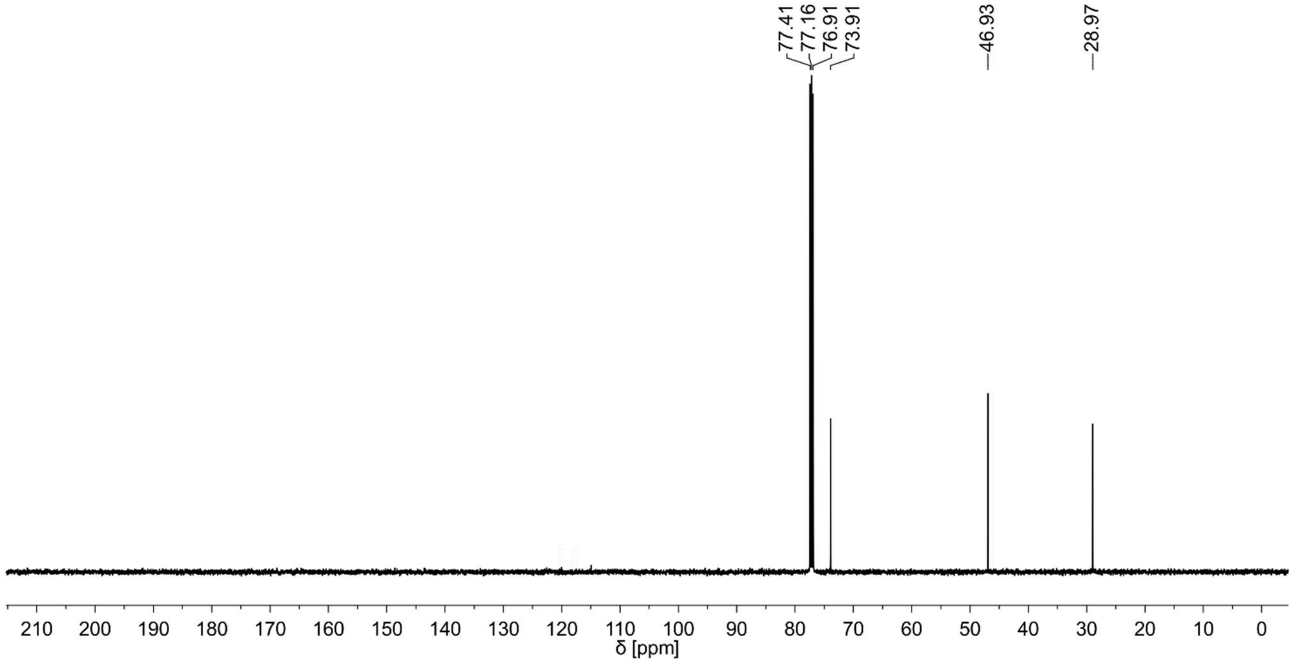

Figure S4. [13]**C-NMR** (126 MHz, $CDCl_3$) of **S6.**

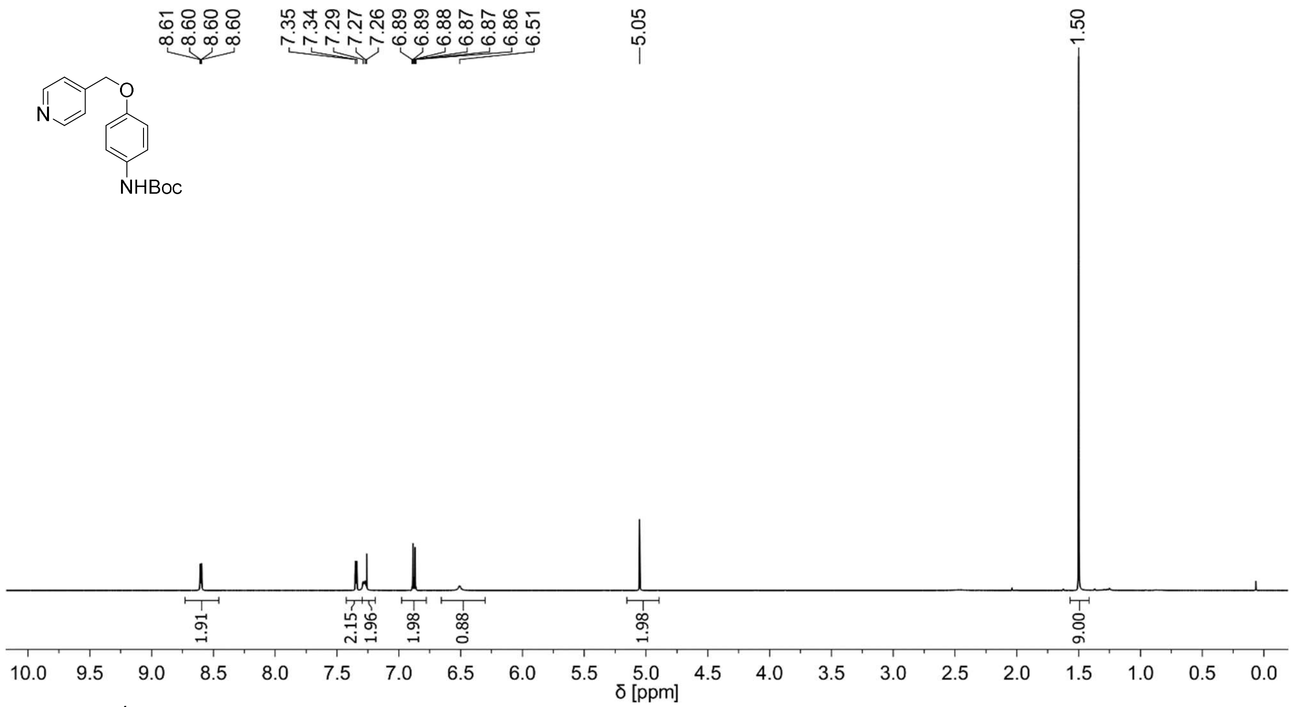

Figure S5. ^{1}H-NMR (500 MHz, $CDCl_3$) of **S3.**

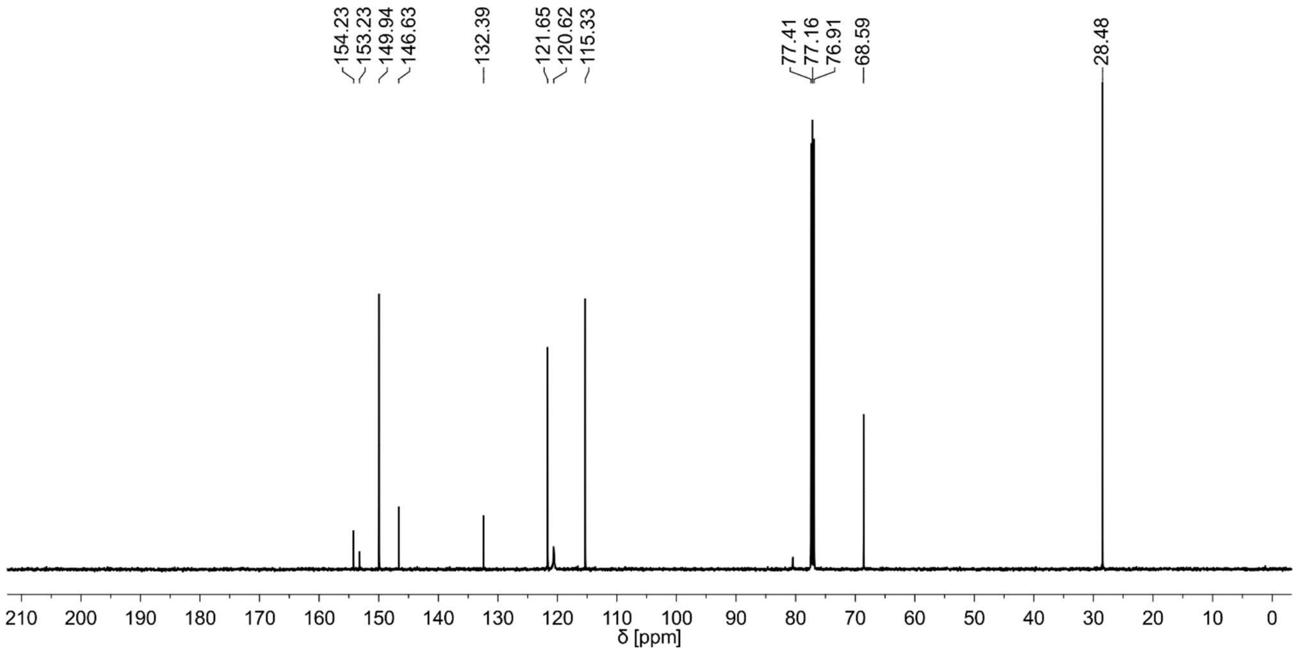

Figure S6. ^{13}C-NMR (126 MHz, $CDCl_3$) of **S3.**

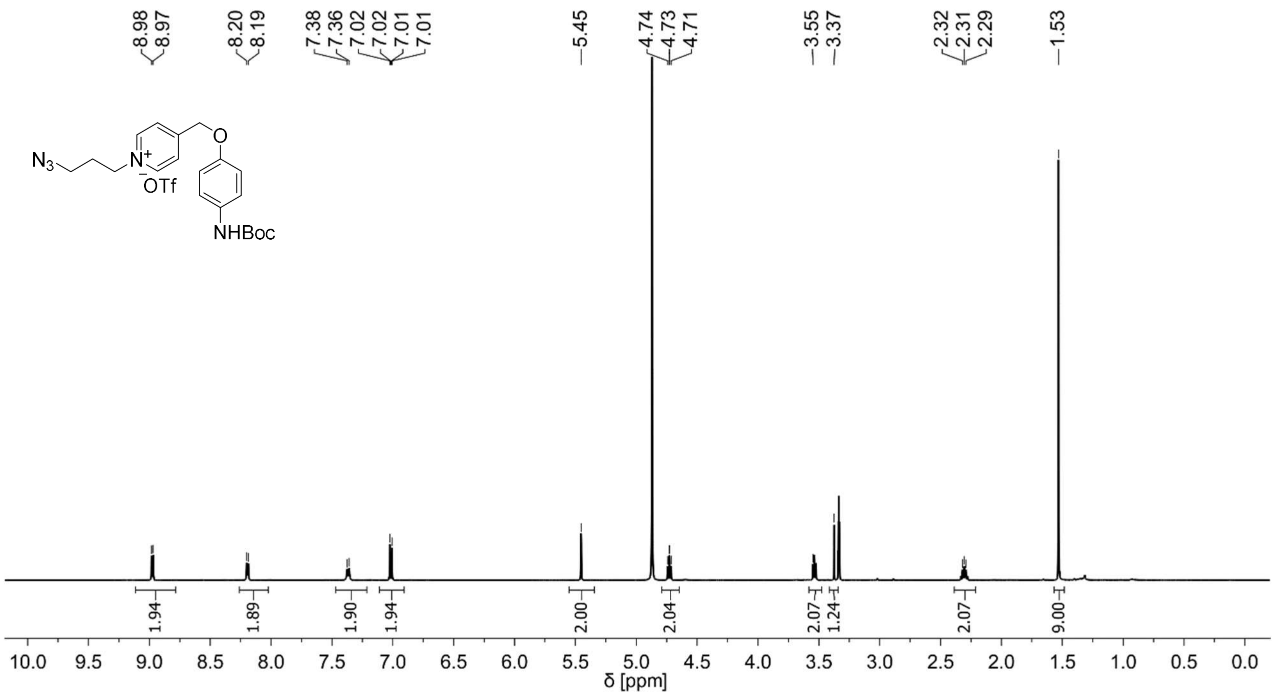

Figure S7. **^{1}H-NMR** (500 MHz, MeOD-d4) of **S7.**

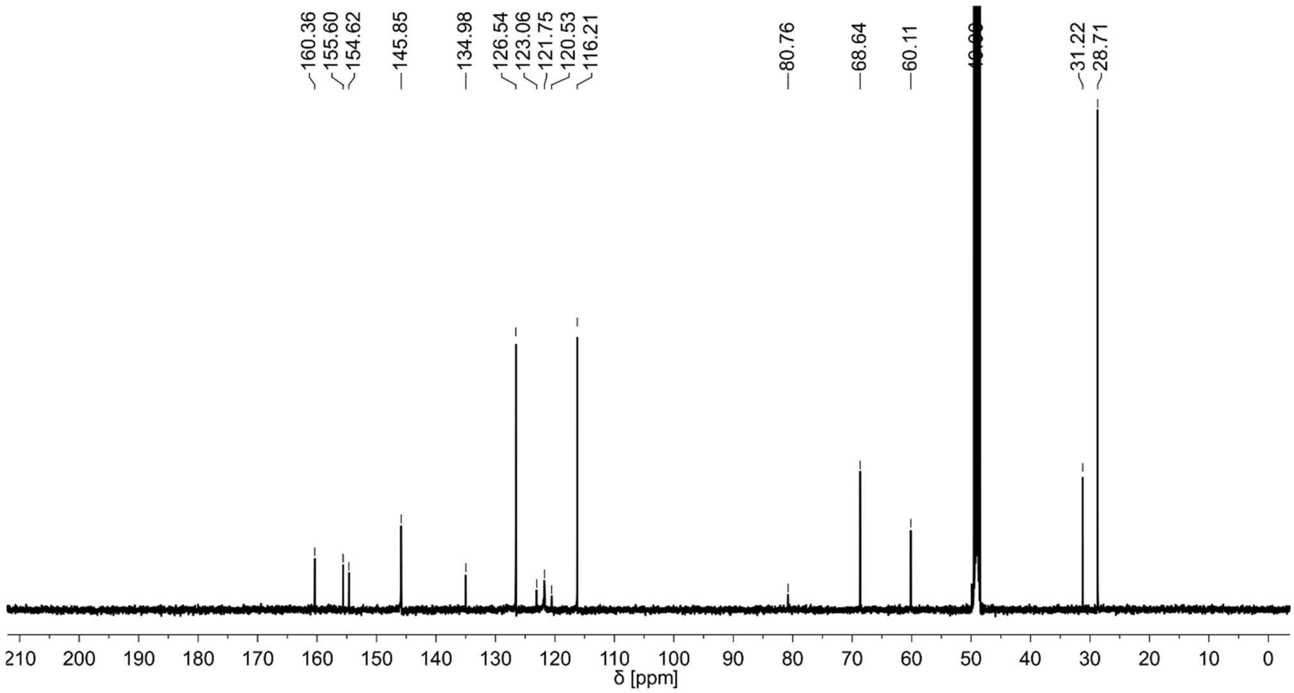

Figure S8. **^{13}C-NMR** (126 MHz, MeOD-d4) of **S7.**

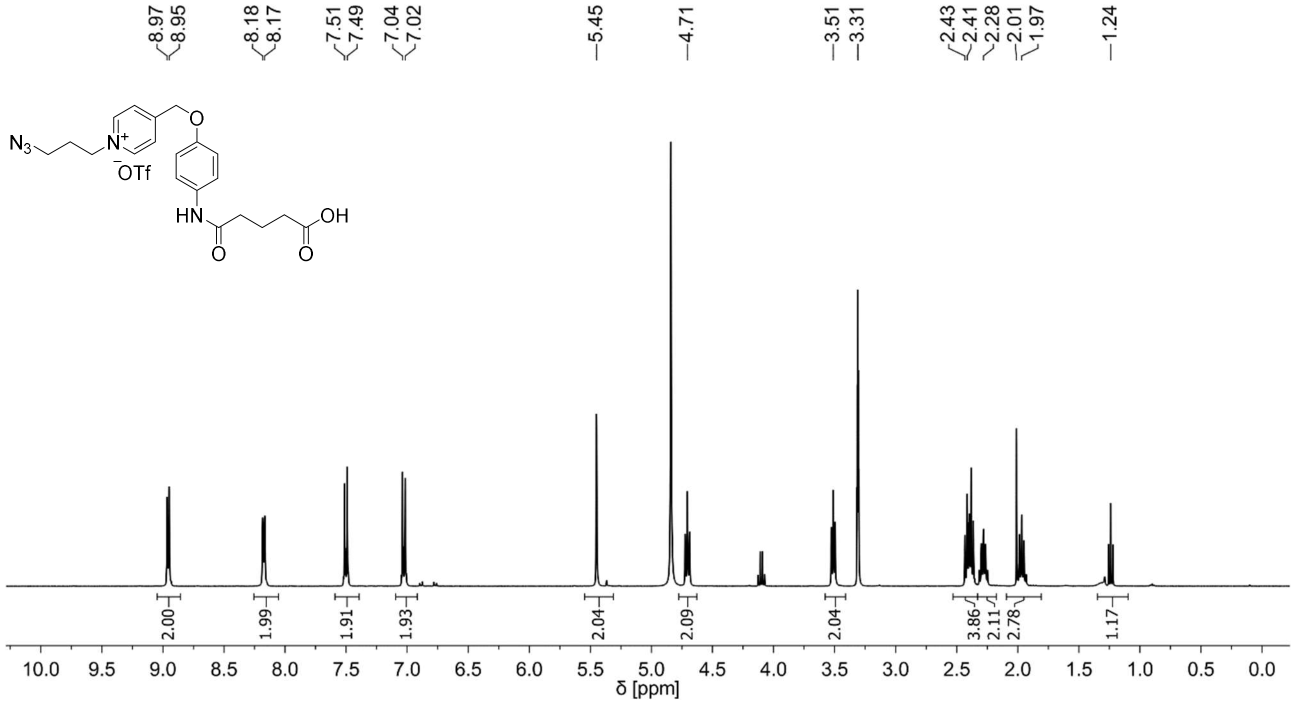

Figure S9. **^{1}H-NMR** (500 MHz, MeOD-d4) of **13.**

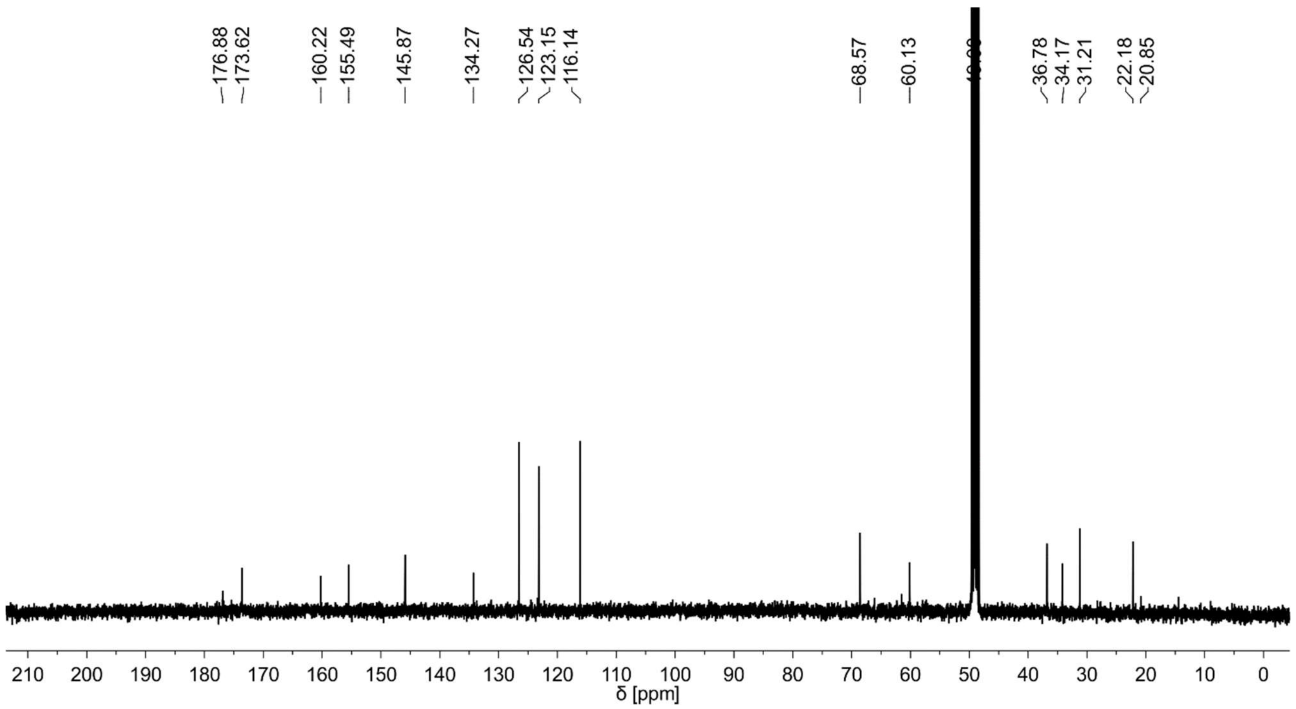

Figure S10. **^{13}C-NMR** (126 MHz, MeOD-d4) of **13.**

2.3 Photolysis of Linker **13** with ATTO425

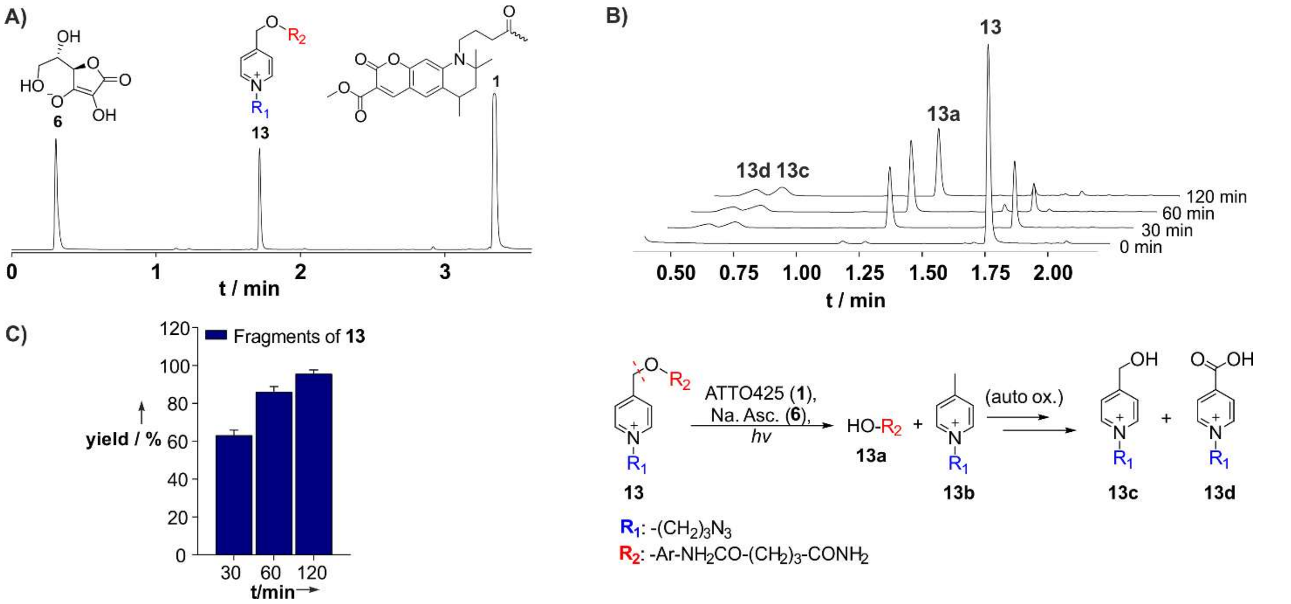

Figure S11. Photo triggered cleavage of **13.** A) UPLC-trace before irradiation and B) close-up after 30, 60 and 120 min; C) calculated yields (integration of peaks). Conditions: 500 µM **13**, 2.5 mM ATTO425 in buffer (10 mM NaH_2PO_4, 100 mM NaCl, 2.5 mM $MgCl_2$, 10 mM Na-Asc., pH = 7.4), irradiation at 455 nm.

2.4 *Turn-on* (50 nM iMB)

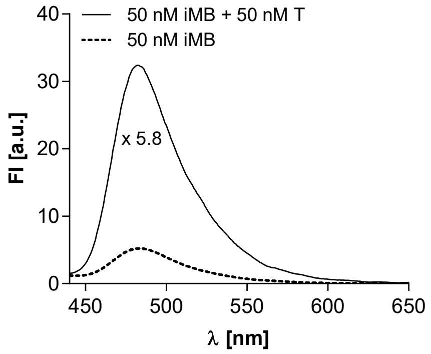

Figure S12. Fluorescence spectra of iMB before (dashed) and after (solid) addition of target T. Conditions: 50 nM iMB, 50 nM T (if added) in PBS buffer (10 mM NaH_2PO_4, 100 mM NaCl, 2.5 mM $MgCl_2$, 5 mM Asc., pH = 7.4), T = 37 °C.

2.5 Photobleaching of ATTO425

A

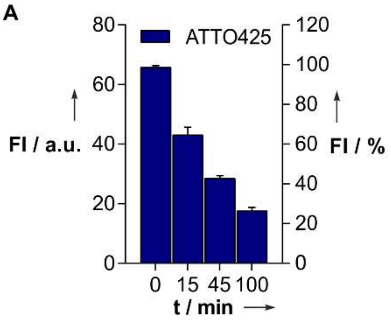

Figure S13. A) Photobleaching of ATTO425 (50 nM) in PBS buffer (10 mM NaH_2PO_4, 100 mM NaCl, 2.5 mM $MgCl_2$, pH = 7.4) upon irradiation at 455 nm for indicated time points. Read out in 1000 µL Quartz cuvettes.

2.6 iMB Synthesis

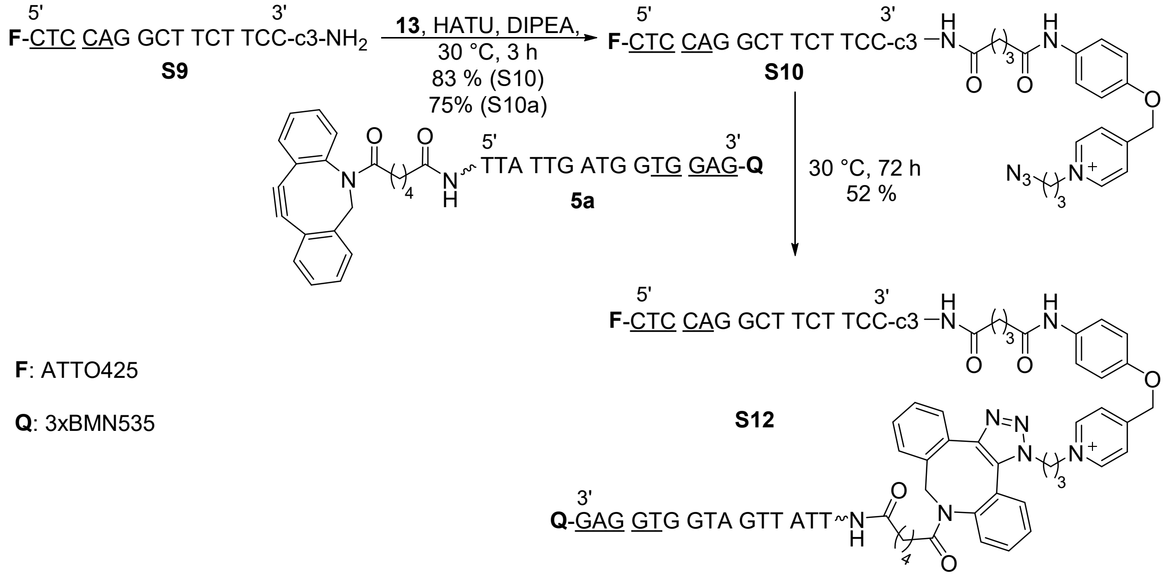

Scheme S2. Synthesis of iMB **S12**

2.6.1 **S10**

A mixture of HATU (10 µmol, 100 eq), DIPEA (30 µmol, 300 eq) and NAP-CL **13** (10 µmol, 100 eq) in DMF (100 uL) was incubated for 4 min. Subsequently, the reaction mixture was transferred to a 2 mL Sarstedt microtube containing oligonucleotide **S9** (100 nmol, 1 eq) in 200 µL water. The mixture was agitated for 3 h at 25 °C. The oligonucleotide was precipitated with iPrOH, vortexed and left for quantitative precipitation at –18 °C for 3 h. After centrifugation (10 min, 13000 rpm), the precipitate was dissolved in 100 µL water. Next, 3M sodium acetate (30 µL) was added and the crude product was precipitated with iPrOH. After another cooling-period at –18 °C for 3 h the crude was centrifuged and dried over Argon. The oligonucleotide was purified via HPLC. The combined fractions were lyophilized and the residue was desalted via precipitation with ammonium acetate and iPrOH.

ATTO425-CTC CAG GCT TCT TCC-ArO-NAP-N_3 (**S10**)

HPLC: t_R = 17 min (10 – 45% B in 25 min)
UPLC: t_R = 2.28 min (3 – 60% B in 4 min)
MALDI-TOF (*m/z*) calcd for: $C_{207}H_{275}N_{58}O_{111}P_{16}$: 5845.35 $[M+H]^+$, found: 5845.0 $[M+H]^+$, 5671.9 $[M+H]^+$ (OH-Fragment). Fragmentation of coupled NAP-CL **13** occurs during MALDI-TOF measurement (laser induced)

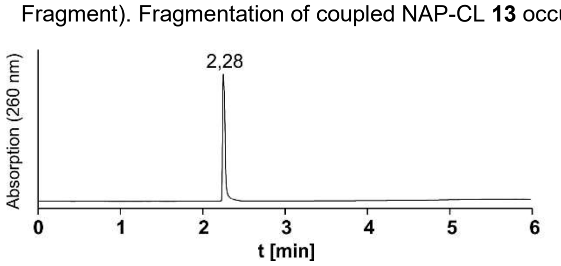

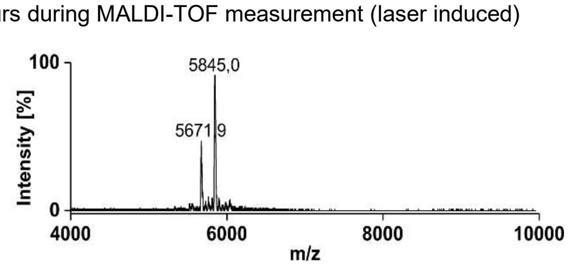

2.6.2 SPAAC (iMB synthesis)

A 2 mL Sarstedt microtube was charged with a solution of **S10** (20 nmol, 1 eq) in a water/ACN (1:1 [v/v], 50 µL) mixture. A solution of DBCO-oligonucleotide (**5a**) (20 nmol, 1.0 – 1.8 eq) in a water/ACN (1:1 [v/v], 50 µL) mixture was added. The solution was agitated for 72 h at 30 °C. Oligonucleotides were precipitated with iPrOH, vortexed and left for quantitative precipitation at –18 °C for 3 h. After centrifugation (10 min, 13000 rpm), the precipitate was dissolved in 100 µL water. Next, 3M sodium acetate (30 µL) was added and the crude product was precipitated with iPrOH. After another cooling-period at –18 °C for 3 h the crude was centrifuged and dried over Argon. The oligonucleotide was purified via HPLC. The combined fractions were lyophilized and the residue was desalted via precipitation with ammonium-acetate and iPrOH.

ATTO425-CTC CAG GCT TCT TCC-ArO-NAP-Triazol-TTA TTG ATG GTG GAC-3xBMN535 (**S12**)

HPLC: t_R = 17 min (25 – 50% B in 25 min, 50% B 15 min, 50 – 75% B in 3 min)

UPLC: t_R = 2.72 min (3 – 80% B in 4 min)
MALDI-TOF (*m/z*) calcd: 12436.2 $[M+H]^+$, found: 12436.3 $[M+H]^+$, 5673.4 $[M+H]^+$ (OH-Fragment). Fragmentation of iMB **S12** occurs during MALDI-TOF measurement (laser induced)

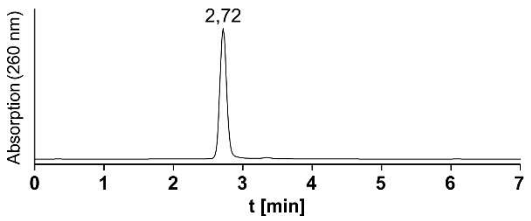

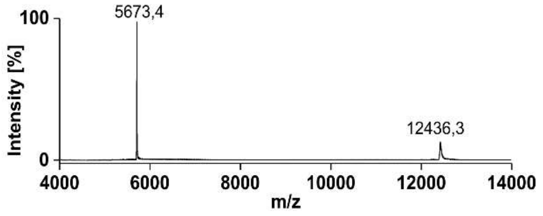

2.6.3 Target-Sequences

Table S1. Sequences of the examined targets. Mismatched nucleobases are highlighted.

Name	Sequence (5' – 3')
**T**	CCA UCA AUA AGG AAG AAG CC
**M_{DNA}**	CCA TCA ATA AGG AAG AAG CC
**1Mm**	CCA UCA AUA AGG **C**AG AAG CC
**2Mm**	CCA **G**CA AUA AGG **C**AG AAG CC
**Mr**	UGC AUC UUG AAA UGU AAU UCG
**M_{1nt}**	CCA UCA AUA A**C**G GAA GAA GCC
**M_{2nt}**	CCA UCA AUA A**CC** GGA AGA AGC C
**M_{3nt}**	CCA UCA AUA A**CG C**GG AAG AAG CC
**M_{4nt}**	CCA UCA AUA A**CG UC**G GAA GAA GCC
**M_{5nt}**	CCA UCA AUA A**CG UCA** GGA AGA AGC C
**M_{6nt}**	CCA UCA AUA A**CG UCA C**GG AAG AAG CC
**M_{7nt}**	CCA UCA AUA A**CG UCA CA**G GAA GAA GCC

2.7 Determination of cleavage yield

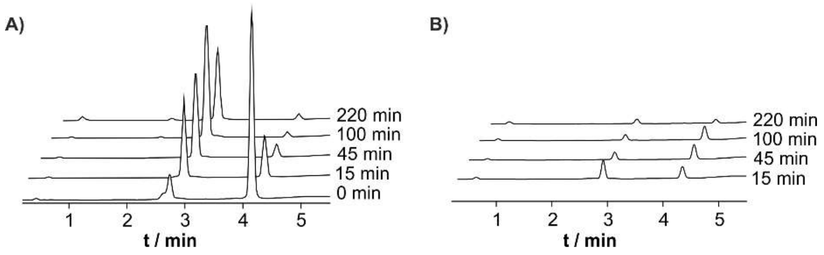

Figure S14. Exemplary UPLC-traces of iMB after photo irradiation in A) presence of 0.1 eq. target T or B) in absence of target. Conditions: 50 nM iMB in PBS buffer (10 mM NaH_2PO_4, 100 mM NaCl, 2.5 mM $MgCl_2$, 5 mM Asc. pH = 7.4), T = 37 °C, irradiation at 455 nm. Traces in A) show the growth of the peak at rt = 2.7 min. This is not observed for traces in B). The rather low signal intensity over time in B) may be due to adsorption.

2.8 Melting (T_M) analysis

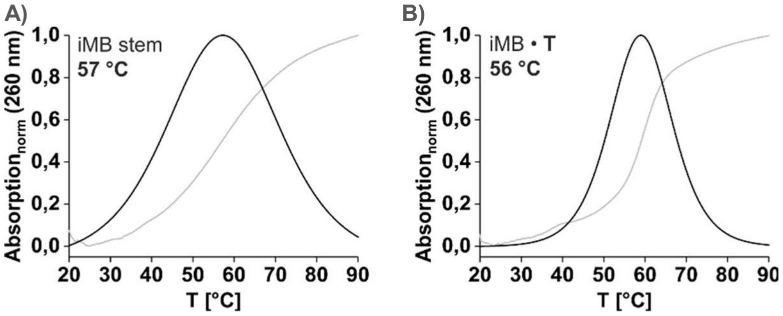

Figure S15. Normalized melting curves of A) iMB alone and B) after addition of 1 eq **T**.

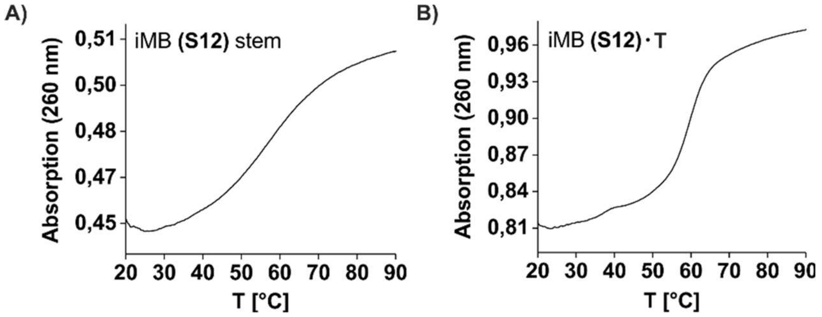

Figure S16. Melting curves of A) iMB alone and B) after addition of 1 eq **T.**

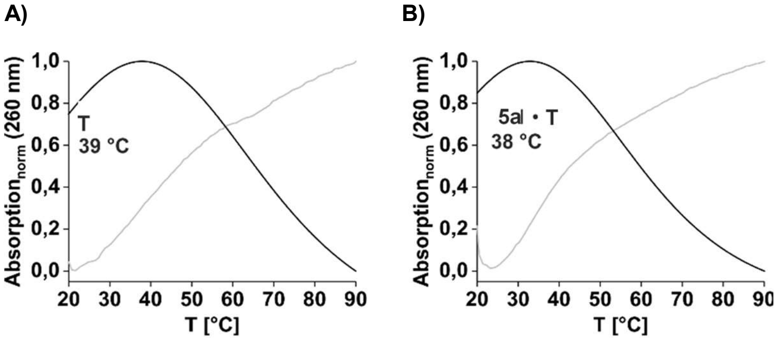

Figure S17. Normalized melting curves of A) **T** and B) **5a · T.**

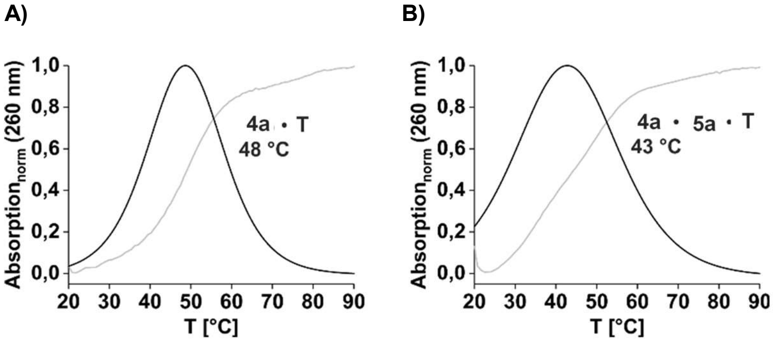

Figure S18. Normalized melting curves of A) **4a · T** and B) **5a · T · 4a**.

2.9 Measurements for limit of detection (LOD)

Table S2. Fluorescence intensities, F/F_0 and signal change (%) upon photo irradiation of the iMB in presence of varied amounts of target **T**. Conditions: 50 nM iMB in PBS buffer (10 mM NaH_2PO_4, 100 mM NaCl, 5 mM Asc., 2.5 mM $MgCl_2$, pH = 7.4), T = 37 °C, irradiation at 455 nm.

eq (T)		0 min	0 min + T		15 min		45 min		100 min		220 min	
		FI / a.u.	FI / a.u.	F/F_0	FI / a.u.	F/F_0	FI / a.u.	F/F_0	FI / a.u.	F/F_0	FI / a.u.	F/F_0
**0**	I	3.10	3.00	0.97	2.58	0.83	2.50	0.81	2.51	0.81	2.50	0.81
	II	3.22	3.22	1.00	2.60	0.81	2.71	0.84	2.84	0.88	2.80	0.87
	III	3.20	3.30	1.03	2.60	0.81	2.66	0.83	2.78	0.87	2.60	0.81
	**avg**	**3.17 ± 0.06**	**3.17 ± 0.16**	**1.00 ± 0.03**	**2.59 ± 0.01**	**0.82 ± 0.01**	**2.62 ± 0.11**	**0.82 ± 0.02**	**2.71 ± 0.18**	**0.85 ± 0.04**	**2.63 ± 0.15**	**0.83 ± 0.03**
**0.002**	I	3.50	3.50	1.00	3.58	1.02	3.90	1.11	4.60	1.31	5.00	1.43
	II	3.50	3.59	1.03	3.59	1.03	3.79	1.08	4.20	1.20	4.96	1.42
	III	3.52	3.52	1.00	3.60	1.02	3.80	1.08	4.50	1.28	4.69	1.33
	**avg**	**3.51 ± 0.01**	**3.54 ± 0.05**	**1.01 ± 0.01**	**3.59 ± 0.01**	**1.02 ± 0.01**	**3.83 ± 0.06**	**1.09 ± 0.02**	**4.43 ± 0.21**	**1.26 ± 0.06**	**4.88 ± 0.17**	**1.97 ± 0.08**
**0.005**	I	4.20	4.35	1.04	5.20	1.24	5.70	1.36	6.20	1.48	6.50	1.55
	II	4.02	4.30	1.07	5.00	1.24	5.70	1.42	6.40	1.59	6.70	1.67
	III	3.70	3.80	1.03	4.30	1.16	4.97	1.34	5.40	1.46	6.30	1.70
	**avg**	**3.97 ± 0.25**	**4.15 ± 0.30**	**1.04 ± 0.02**	**4.83 ± 0.47**	**1.21 ± 0.05**	**5.46 ± 0.42**	**1.37 ± 0.04**	**6.00 ± 0.53**	**1.51 ± 0.07**	**6.50 ± 0.20**	**1.64 ± 0.08**
**0.01**	I	3.35	3.55	1.06	4.92	1.47	5.90	1.76	6.24	1.86	7.40	2.21
	II	3.42	3.68	1.08	4.59	1.34	5.34	1.56	6.05	1.77	7.69	2.25
	III	3.40	3.70	1.09	4.14	1.22	4.90	1.44	5.22	1.54	5.20	1.54
	**avg**	**3.39 ± 0.04**	**3.64 ± 0.08**	**1.07 ± 0.05**	**4.55 ± 0.39**	**1.34 ± 0.13**	**5.38 ± 0.50**	**1.59 ± 0.16**	**5.84 ± 0.54**	**1.72 ± 0.17**	**6.76 ± 1.36**	**1.72 ± 0.40**
**0.02**	I	3.10	3.56	1.15	5.00	1.61	6.15	1.98	6.58	2.12	7.40	2.39
	II	3.47	3.82	1.10	4.87	1.40	5.94	1.71	6.89	1.99	9.00	2.59
	III	3.10	3.38	1.09	4.30	1.39	5.00	1.61	5.38	1.74	5.40	1.74
	**avg**	**3.22 ± 0.21**	**3.59 ± 0.22**	**1.11 ± 0.03**	**4.72 ± 0.37**	**1.47 ± 0.13**	**5.67 ± 0.61**	**1.77 ± 0.19**	**6.28 ± 0.79**	**1.95 ± 0.20**	**7.27 ± 1.80**	**2.24 ± 0.44**
**0.1**	I	4.10	6.37	1.55	8.20	2.00	10.17	2.48	9.66	2.36	10.02	2.44
	II	3.37	5.00	1.48	6.90	2.05	8.40	2.49	8.70	2.58	8.40	2.49
	III	3.80	5.77	1.52	7.51	1.98	9.31	2.45	9.38	2.47	9.40	2.47
	**avg**	**3.76 ± 0.37**	**5.71 ± 0.69**	**1.52 ± 0.03**	**7.54 ± 0.65**	**2.01 ± 0.04**	**9.25 ± 0.49**	**2.47 ± 0.02**	**9.25 ± 0.49**	**2.47 ± 0.11**	**9.27 ± 0.82**	**2.47 ± 0.02**
**1.0**	I	4.50	25.70	5.71								
	II	4.80	25.60	5.33								
	III	5.10	33.10	6.49								
	**avg**	**4.80 ± 0.30**	**28.13 ± 4.30**	**5.84 ± 0.59**								

Table S3. Fluorescence intensities, F/F_0 and signal change (%) upon photo irradiation of the iMB in presence of varied amounts of target **T**. Conditions: 50 nM iMB in PBS buffer (10 mM NaH_2PO_4, 100 mM NaCl, 5 mM Asc., 2.5 mM $MgCl_2$, 0.001% Tween20, pH = 7.4), T = 37 °C, irradiation at 455 nm.

**eq (T)**		**0 min**	**0 min + T**		**15 min**		**45 min**		**100 min**		**220 min**	
		**FI / a.u.**	**FI / a.u.**	**F/F_0**	**FI / a.u.**	**F/F_0**	**FI / a.u.**	**F/F_0**	**FI / a.u.**	**F/F_0**	**FI / a.u.**	**F/F_0**
**0**	I	3.66	3.66	1.00	3.56	0.97	4.10	1.12	4.48	1.22	5.90	1.61
	II	3.70	3.70	1.00	3.60	0.97	4.61	1.24	4.68	1.26	5.10	1.38
	III	3.70	3.70	1.00	3.00	0.81	3.70	1.00	4.40	1.19	5.20	1.41
	**avg**	**3.69 ± 0.02**	**3.69 ± 0.02**	**1.00 ± 0**	**3.39 ± 0.34**	**0.92 ± 0.09**	**4.13 ± 0.45**	**1.12 ± 0.12**	**4.52 ± 0.14**	**1.22 ± 0.04**	**5.40 ± 0.44**	**1.47 ± 0.13**
**0.0001**	I	3.50	3.50	1.00	3.80	1.09	4.60	1.31	5.60	1.60	6.00	1.71
	II	3.81	3.81	1.00	4.08	1.07	4.77	1.26	5.50	1.45	6.00	1.58
	III	3.80	3.75	0.99	3.85	1.01	4.70	1.24	5.70	1.50	6.70	1.76
	**avg**	**3.70 ± 0.17**	**3.68 ± 0.16**	**1.00 ± 0.01**	**3.91 ± 0.15**	**1.06 ± 0.04**	**4.69 ± 0.09**	**1.27 ± 0.04**	**5.60 ± 0.10**	**1.52 ± 0.08**	**6.23 ± 0.40**	**1.69 ± 0.10**
**0.0002**	I	3.50	3.50	1.00	3.80	1.09	4.81	1.37	5.60	1.60	6.00	1.71
	II	3.80	3.85	1.01	4.13	1.09	4.92	1.30	5.70	1.50	6.20	1.63
	III	3.76	3.81	1.01	4.20	1.12	5.10	1.36	6.01	1.60	6.70	1.78
	**avg**	**3.69 ± 0.16**	**3.72 ± 0.19**	**1.01 ± 0.01**	**4.04 ± 0.21**	**1.10 ± 0.02**	**4.94 ± 0.15**	**1.34 ± 0.04**	**5.77 ± 0.21**	**1.57 ± 0.06**	**6.30 ± 0.36**	**1.71 ± 0.08**
**0.0005**	I	3.50	3.50	1.00	4.07	1.16	4.76	1.36	5.90	1.69	6.20	1.77
	II	4.04	4.04	1.00	4.60	1.14	5.77	1.43	6.89	1.71	6.90	1.71
	III	3.80	3.80	1.00	4.30	1.13	5.30	1.39	6.20	1.63	7.20	1.89
	**avg**	**3.78 ± 0.27**	**3.78 ± 0.27**	**1.00 ± 0**	**4.32 ± 0.27**	**1.14 ± 0.02**	**5.28 ± 0.51**	**1.39 ± 0.03**	**6.33 ± 0.51**	**1.67 ± 0.04**	**6.77 ± 0.51**	**1.79 ± 0.09**
**0.001**	I	3.60	3.69	1.03	4.20	1.17	5.24	1.46	6.67	1.85	7.40	2.06
	II	4.20	4.13	0.98	4.90	1.17	6.17	1.47	7.89	1.88	8.20	1.95
	III	3.87	4.10	1.06	4.61	1.19	5.78	1.49	6.70	1.73	7.37	1.90
	**avg**	**3.89 ± 0.30**	**3.97 ± 0.25**	**1.02 ± 0.04**	**4.57 ± 0.35**	**1.17 ± 0.01**	**5.73 ± 0.47**	**1.47 ± 0.02**	**7.09 ± 0.70**	**1.82 ± 0.08**	**7.66 ± 0.47**	**1.97 ± 0.08**

2.10 Specificity of iMB signalling

Table S4. Fluorescence intensities, F/F_0 and signal change (%) afforded by iMB prior to and 15 or 100 min after photoirradiation in presence of mismatched RNA. Conditions: 50 nM iMB, 5 nM RNA mix (**2Mm**, **Mr**, **M_{7nt}**), PBS buffer (10 mM NaH_2PO_4, 100 mM NaCl, 2.5 mM $MgCl_2$, 5 mM Asc. pH = 7.4), T = 37 °C, irradiation at 455 nm.

**eq (T)**		**0 min**	**0 min + T**			**15 min**			**100 min**		
		**FI / a.u.**	**FI / a.u.**	**F/F_0**	**Signal change / %**	**FI / a.u.**	**F/F_0**	**Signal change / %**	**FI / a.u.**	**F/F_0**	**Signal change / %**
**0**	I	6.20	6.40	1.03	3.23	5.78	0.93	-6.77	6.65	1.07	7.26
	II	6.50	6.20	0.95	-4.61	5.30	0.81	-18.46	6.20	0.86	-4.62
	III	5.80	6.00	1.03	3.44	5.70	0.87	-12.59	6.60	0.98	13.79
	**avg**	**6.12 ± 0.35**	**6.20 ± 0.20**	**1.01 ± 0.05**	**0.67 ± 4.59**	**5.38 ± 0.36**	**0.87 ± 0.06**	**-12.61 ± 5.84**	**6.48 ± 0.25**	**0.96 ± 0.09**	**5.48 ± 9.33**
**0.0025**	I	6.10	6.58	1.08	7.87	6.12	1.00	0.33	6.85	1.12	12.30
	II	5.78	5.78	1.00	0	6.08	1.05	5.19	7.90	1.37	36.68
	III	5.60	5.70	1.02	1.79	5.80	1.04	3.57	6.18	1.10	10.36
	**avg**	**5.83 ± 0.25**	**6.02 ± 0.49**	**1.03 ± 0.04**	**3.22 ± 4.13**	**6.00 ± 0.17**	**1.03 ± 0.02**	**3.03 ± 2.48**	**6.98 ± 0.87**	**1.20 ± 0.15**	**19.78 ± 14.67**
**0.005**	I	5.35	5.90	1.10	10.28	6.24	1.17	16.64	7.72	1.44	44.30
	II	5.87	6.10	1.04	3.92	6.70	1.14	14.14	7.80	1.33	32.88
	III	6.10	6.70	1.10	9.84	6.70	1.10	9.84	7.00	1.15	14.75
	**avg**	**5.77 ± 0.38**	**6.23 ± 0.42**	**1.08 ± 0.04**	**8.01 ± 3.55**	**6.55 ± 0.27**	**1.14 ± 0.03**	**13.54 ± 3.44**	**7.51 ± 0.44**	**1.15 ± 0.08**	**14.63 ± 7.54**
**0.01**	I	5.88	6.88	1.17	17.01	8.88	1.51	51.02	9.20	1.56	56.46
	II	6.59	7.70	1.17	16.84	9.60	1.46	51.90	10.13	1.54	53.72
	III	6.49	7.50	1.16	15.56	9.78	1.51	57.94	10.20	1.57	57.16
	**avg**	**6.32 ± 0.38**	**7.36 ± 0.43**	**1.16 ± 0.01**	**16.47 ± 0.79**	**9.42 ± 0.48**	**1.49 ± 0.03**	**54.86 ± 3.02**	**9.84 ± 0.56**	**1.56 ± 0.02**	**55.78 ± 1.82**
**0.1**	I	6.50	9.01	1.39	38.62	11.45	1.76	76.15	14.80	2.28	127.69
	II	6.80	9.30	1.37	36.76	11.70	1.72	72.06	15.38	2.26	126.18
	III	6.24	9.10	1.46	45.83	11.10	1.78	77.88	15.50	2.48	148.40
	**avg**	**6.50 ± 0.28**	**9.14 ± 0.15**	**1.40 ± 0.05**	**40.40 ± 4.79**	**11.41 ± 0.30**	**1.75 ± 0.03**	**75.36 ± 3.00**	**15.23 ± 0.37**	**2.34 ± 0.12**	**134.09 ± 12.41**

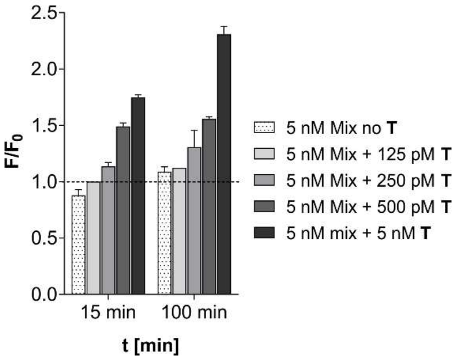

Figure S19. Signal change afforded by **iMB** upon photo irradiation in presence of mismatched RNA. Graphical representation of data in Table S4

2.11 Measurements in RNA-extract and cell Lysate (HEK293WT)

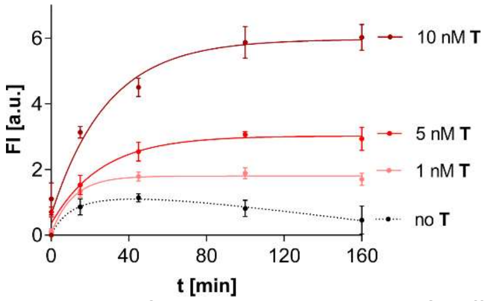

Figure S20. Signalling from the iMB in RNA extract. Conditions: 100 nM iMB, PBS buffer (10 mM NaH_2PO_4, 100 mM NaCl, 5 mM Asc., 0.001% Tween20, pH = 7.4), T = 37 °C, irradiation at 455 nm. The reaction was carried out in 96 well plates, V_{react}: 150 µL, read-out: 100 µL suprasil ultra-micro quartz cuvettes.

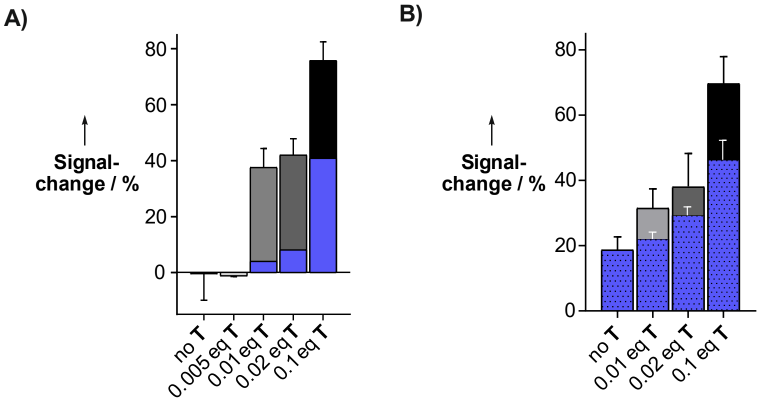

Figure S21. Signal change upon photo irradiation (15 min) of iMB in A) cell lysate or B) RNA extract spiked with indicated equivalents of target **T**. The blue overlay indicates the theoretical signal change provided by a non-cleavable MB probe with similar fluorescence turn-on characteristics. Conditions: A) 50 nM iMB in 8 vol% lysate from 10 mio. HEK293 in PBS buffer containing 5 mM $MgCl_2$, 5 mM Asc., pH = 7.4, 37 °C. Read out in 1000 µL suprasil quartz cuvettes. B) 100 nM iMB in RNA extract (5 µg in PBS buffer, 5 mM Asc., 0.001% Tween20, pH = 7.4) at 37 °C. Read-out in 100 µL suprasil ultra-micro quartz cuvettes.

Table S5. Measurements in cell lysate. Fluorescence Intensities (a.u.); c (iMB) = 50 nM, PBS-buffer (10 mM NaH_2PO_4, 100 mM NaCl, 5 mM Asc., 5 mM $MgCl_2$, pH = 7.4), T = 37 °C, irradiation at 455 nm.

eq (T)		0 min	0 min + T		15 min		45 min		100 min	
		FI / a.u.	FI / a.u.	F/F$_0$	FI / a.u.	F/F$_0$	FI / a.u.	F/F$_0$	FI / a.u.	F/F$_0$
**0**	I	4.72	4.57	0.97	3.84	0.81	3.80	0.81	4.50	0.95
	II	4.65	4.67	1.00	5.26	1.13	5.84	1.26	6.00	1.29
	III	5.10	5.10	1.00	5.30	1.04	6.10	1.20	5.90	1.16
	avg	**4.82 ± 0.24**	**4.78 ± 0.28**	**1.00 ± 0.02**	**4.80 ± 0.28**	**1.00 ± 0.16**	**5.25 ± 1.26**	**1.09 ± 0.24**	**5.47 ± 0.84**	**1.14 ± 0.17**
**0.005**	I	4.94	5.20	1.05	4.88	0.99	5.30	1.07	5.40	1.09
	II	4.57	4.94	1.08	4.50	0.98	4.83	1.06	4.99	1.09
	III	4.74	5.05	1.07	4.69	0.99	5.07	1.07	5.21	1.10
	avg	**4.75 ± 0.19**	**4.69 ± 0.19**	**1.07 ± 0.01**	**4.69 ± 0.19**	**0.99 ± 0.01**	**5.07 ± 0.24**	**1.07 ± 0.01**	**5.20 ± 0.21**	**1.09 ± 0.01**
**0.01**	I	5.49	6.02	1.10	7.43	1.35	7.53	1.37	6.60	1.20
	II	4.94	5.54	1.12	7.17	1.45	6.96	1.41	5.97	1.21
	III	4.97	5.55	1.12	6.57	1.32	6.76	1.36	6.40	1.29

eq (T)		0 min	0 min + T		15 min		45 min		100 min	
		FI / a.u.	FI / a.u.	F/F_0	FI / a.u.	F/F_0	FI / a.u.	F/F_0	FI / a.u.	F/F_0
	avg	**5.13 ± 0.31**	**5.70 ± 0.27**	**1.11 ± 0.01**	**7.06 ± 0.44**	**1.38 ± 0.07**	**7.08 ± 0.40**	**1.38 ± 0.03**	**6.23 ± 0.32**	**1.23 ± 0.05**
**0.02**	I	4.83	5.65	1.17	7.18	1.49	7.30	1.51	7.00	1.45
	II	5.29	5.88	1.11	7.30	1.38	7.30	.138	6.70	1.27
	III	5.49	6.41	1.17	7.64	1.39	8.05	1.47	8.22	1.50
	avg	**5.20 ± 3.30**	**5.98 ± 0.39**	**1.14 ± 0.03**	**7.37 ± 0.24**	**1.42 ± 0.06**	**7.55 ± 0.43**	**1.45 ± 0.07**	**7.31 ± 0.81**	**1.40 ± 0.12**
**0.1**	I	5.61	8.02	1.43	9.43	1.68	9.88	1.76	10.26	1.83
	II	5.34	7.90	1.48	9.55	1.79	9.66	1.81	9.70	1.82
	III	5.36	8.00	1.49	9.66	1.80	11.05	2.06	12.27	2.29
	avg	**5.44 ± 0.15**	**7.97 ± 0.06**	**1.47 ± 0.03**	**9.55 ± 0.12**	**1.76 ± 0.07**	**10.20 ± 0.75**	**1.88 ± 0.16**	**10.74 ± 1.35**	**1.98 ± 0.27**

References

[1] S. Kalhor-Monfared, C. Beauvineau, D. Scherman, C. Girard, *Eur. J. Med. Chem.* **2016**, *122*, 436–441.
[2] J. R. Kramer, T. J. Deming, *Biomacromolecules* **2012**, *13*, 1719–1723.
